# Adapting a safe water storage container to improve household stored water quality in rural Burkina Faso: a cluster randomized trial

**DOI:** 10.2166/washdev.2021.065

**Published:** 2021-08-10

**Authors:** Darcy M. Anderson, Michael B. Fisher, Osborn Kwena, Hermann Kambou, Romain Broseus, Ashley R. Williams, Kaida Liang, Rohit Ramaswamy, Jamie Bartram

**Affiliations:** a The Water Institute at UNC, University of North Carolina at Chapel Hill, Chapel Hill, NC, USA; b WaterAid, Ouagadougou, Burkina Faso; c WaterAid, New York City, NY, USA; d ICF, Durham, NC, USA; e Gillings School of Global Public Health, University of North Carolina at Chapel Hill, Chapel Hill, NC, USA; f School of Civil Engineering, University of Leeds, Leeds, UK

**Keywords:** adaptation, Burkina Faso, drinking water, *Escherichia coli*, Plan-Do-Study-Act, safe storage

## Abstract

Safe water storage protects household drinking water from microbial contamination, maintaining water quality and preventing diarrhea and other water-borne diseases. However, achieving high adoption and sustained use of safe storage is challenging. Systematic adaptation can address these challenges by improving contextual fit while retaining core functionality to protect water quality. We applied Plan-Do-Study-Act (PDSA) cycles to systematically adapt a safe water storage container (SWSC) intervention for implementation in rural Burkina Faso. This study describes the adaptation process and the impacts of the SWSC on *Escherichia coli* contamination in household stored water in a cluster-randomized trial with 49 intervention villages (274 households) and 50 no-intervention control villages (290 households). SWSC adoption among intervention households was high (88.9%). The intervention achieved approximately a 0.4 log reduction in *E. coli* contamination. Intervention impact was likely moderated by differential changes in improved source use across intervention and control households. Safe storage improves water quality when used consistently. PDSA frameworks can guide the adaptation of safe storage interventions to optimize adoption and sustained use in new contexts while preserving core functions that protect water quality.

## INTRODUCTION

Access to safe drinking water is an important predictor of health. Unsafe drinking water is associated with various adverse health outcomes, including diarrheal disease and other enteric infections (Bartram & Cairncross 2010; Clasen *et al*. 2015). Approximately 485,000 deaths annually from diarrheal disease are attributable to unsafe drinking water in low- and middle-income countries (Prüss-Ustün *et al*. 2019). Repeated enteric infections can result in environmental enteropathy, causing long-term and sometimes permanent impairments to physical growth and cognitive development (Guerrant *et al*. 2012).

Goal 6 under the sustainable development goals sets targets for achieving ‘safely managed’ water for all, where safely managed is defined as a source ‘located on the premises, available when needed, and free of fecal and priority chemical contamination’ (WHO 2017). In contrast, ‘basic’ services comprise access to a water source that is located within a 30-min round-trip collection time and is protected from contamination due to its construction or design but not tested for chemical or microbial safety. Estimated 1.4 billion people rely on basic services, while further 206 million use a protected source requiring greater than 30-min collection time (WHO/UNICEF 2020).

Off-site and intermittent sources typically necessitate storage of water within the home. Collection and storage are potential entry points for fecal contamination from sources that are otherwise safe, as unclean hands, containers, or other surfaces can introduce microbial contamination (Wright *et al*. 2004). Safe water storage is defined as storing drinking water in a container with a tight-fitting lid and a narrow mouth to prevent the introduction of unwashed hands and extracting water by pouring or using a tap. Safe water storage interventions can decrease water contamination risk (Mintz *et al*. 1995; Wright *et al*. 2004). However, interventions to improve household stored water (HSW) quality have struggled to achieve and sustain high levels of uptake and use, and overcoming habitual practices to change household water storage has proved challenging (Lantagne *et al*. 2006; Murcott 2006; Sobsey *et al*. 2008; Parker Fiebelkorn *et al*. 2012).

Overcoming these challenges requires adapting interventions to suit local preferences and production capabilities while preserving core functions (i.e., containers’ ability to block the introduction of contaminants) (Lee *et al*. 2008; Perez Jolles *et al*. 2019; Kirk *et al*. 2020). Plan-Do-Study-Act (PDSA) cycles are a structured process for defining target outcomes for adaptation (plan), executing changes (do), assessing impacts (study), and scaling up effective changes (act). PDSA cycles for structured adaptation have been effective in the manufacturing and health-care sectors (Nicolay *et al*. 2012; Taylor *et al*. 2014) but have rarely been applied to improving water, sanitation, and hygiene (WaSH) interventions in low- and middle-income countries (Fisher *et al*. 2016).

In this study, we present the systematic adaptation of a safe water storage container (SWSC) using PDSA cycles. The original SWSC was first designed and implemented in Ghana (Fisher *et al*. 2020), and here we describe its adaption for use in rural Burkina Faso. We describe the systematic adaptation process and evaluate the adapted SWSC intervention’s impact on the microbial quality of HSW for consumption in a cluster randomized trial.

This trial contributes to the body of literature on SWSC interventions by exploring PDSA as an option to improve the uptake and contextual fit of an intervention that has been proved to protect water quality in other settings. Multiple studies have evaluated the impact of SWSCs on water quality and health (Clasen *et al*. 2015), but to our knowledge, this is the first trial to describe PDSA as a potential tool to adapt WaSH interventions in low-income settings. We propose that our results demonstrate PDSA as a viable tool to improve contextual fit while preserving the functionality to protect against pathogen exposure.

## METHODS

### Study design

Intervention delivery took place in six regions of Burkina Faso (Boucle du Mouhoun, Centre, Centre-Est, Centre-Ouest, Nord, and Sud-Ouest). Data collection occurred between September 2015 and January 2016 at baseline and between February and May 2018 at end line. Intervention delivery was done by WaterAid Burkina Faso, with support from the Water Institute at the University of North Carolina at Chapel Hill (UNC).

One hundred villages were randomly selected from a sampling frame of all villages in which WaterAid worked (*n* = 401). Selected villages were randomly allocated to intervention or control arms using a random number generator in a ratio of 1:1. Within each selected village, all households were enumerated and six were randomly selected to participate in the study. Sample size was determined based on 80% power to detect a 20% reduction (85–65%) in the proportion of households with detectable *E. coli* in a 100-mL water sample. Baseline prevalence *E. coli* and the design effect of 2.0 were estimated based on data from the Ghana trial and confirmed by formative research in Burkinabe communities. We assumed a 15% loss to the follow-up of households, for a sample size of 26 villages per arm, with 6 households per village. The sample size was increased to 50 villages per arm to provide adequate power to detect differences in source characteristics, functionality, and other chemical water quality outcomes of water sources at the community level, which are beyond the scope of this study but have been reported in part elsewhere (Fisher *et al*. 2021).

The primary outcome was *E. coli* contamination in HSW. We assessed confounding by point-of-use water treatment, soap presence, household self-reported open defecation, rainfall, and source water quality (Kostyla *et al*. 2015). This trial was unblinded, as the protective mechanism of SWSCs is large, visible infrastructure components.

### Intervention design and adaptation process

The implementation of the SWSC intervention was done by WaterAid Burkina Faso with support from UNC Chapel Hill. WaterAid is an international organization with a mission to transform the lives of the poorest and most marginalized people by improving access to safe WaSH. WaterAid has been delivering WaSH programming in Burkina Faso since 2001. Prior to the start of this study, WaterAid Burkina Faso conducted formative research and a WaSH needs assessment across its program areas. This needs assessment indicated high access to safe water sources but low household water quality, and as such WaterAid prioritized SWSCs as the target intervention.

The SWSC intervention had been initially developed as part of a previously reported continuous quality improvement (CQI) project in Ghana (Fisher *et al*. 2020). In brief, the Ghanaian intervention had been developed using the Lean Six Sigma CQI framework (Pyzdek & Keller 2014). This framework comprises an iterative cycle of five steps to define performance targets, measure performance, analyze factors contributing to poor performance, design improvement solutions, and verify that the improvements lead to sustainable results. In Ghana, Lean Six Sigma had been adapted to include a sixth step (implement), and the process was used to design SWSCs that met user preferences, conformed to guidelines for SWSC design (CDC 2012), and increased the proportion of households with HSW conforming to World Health Organization (WHO) guidelines for microbial water safety (WHO 2011). The Ghanaian intervention included SWSCs and behavior change messaging on container use, cleaning, and source selection with an emphasis on using safe water sources (i.e., those meeting criteria for ‘basic’ water service).

In this study, we systematically adapted SWSCs developed in Ghana to the Burkinabe context, using PDSA cycles. PDSA cycles are a component of the Lean Six Sigma methodology described above and are small-scale iterative experiments to test changes to the design of a product or process. A team of implementers from WaterAid Burkina Faso were trained on Lean Six Sigma methods by the UNC Water Institute using a manual developed for the Ghana study that customized Lean Six Sigma for WaSH applications (Fisher *et al*. 2017). The adaptation process in Burkina Faso focused on changing SWSCs to meet local preferences and manufacturing capabilities. Messaging on SWSC cleaning, appropriate use, and safe water source selection was translated to local languages, but was otherwise not substantively changed from the Ghana implementation.

To inform the adaptation, assessments were made on three implementation outcomes (acceptability, feasibility, and cost) (Proctor *et al*. 2011) and microbial HSW quality (*E. coli* concentration). To assess acceptability, focus groups were held with households to understand their water collection and storage practices and preferences. Participants were presented with several SWSC designs from Ghana and locally available alternatives. For each option, participants described their likes, dislikes, and likelihood of purchase and use. To assess feasibility and cost, local artisans were presented with the Ghana SWSC designs and interviewed about their ability to manufacture and prices for each design. Focus group and interview guides are included in Supplementary Material, Files SI-1 and SI-2, respectively.

Following community and artisan feedback, the Ghana designs were rated by WaterAid with support from the UNC Water Institute on their ability to protect water quality, cost, and usability using a Pugh matrix, a decision-making tool that uses weighted performance criteria to compare solutions (Pyzdek & Keller 2014). The Pugh matrix considered the following variables: water quality protection, cost, acceptability (user preferences, ease of cleaning and maintenance, water temperature, container volume), and feasibility (ease of manufacturing durability). Each container design was scored using the Pugh matrix, and the highest-scoring container was selected. These variables were selected to balance the ability of the SWSC to protect water quality with the goal of achieving high adoption in recipient households.

Based on these assessments, the Ghanaian SWSC design with adapted taps to improve flow rate was selected as the first Burkinabe prototype. This prototype was tested in a subset of 12 intervention villages, which were selected by convenience based on their proximity and accessibility from the capital. Surveys monitored the household use of containers, reasons for non-use where applicable, and preferences about container design. The data from these surveys were used to conduct the PDSA cycles for adaptation. HSW water quality was tested as described below. Supplementary Material, File SI_3 contains the questionnaire used to assess container use and user preferences during prototype testing.

Following the first round of testing, SWSCs were modified to improve the fit of container lids and to redesign the interior guard (a piece fitted to the container opening to prevent dipping and scooping) to make SWSCs easier to clean. The refined design was implemented in further 13 intervention villages and evaluated as above. No further refinements were made following the second round of testing, and the SWSC was implemented at a scale in all intervention villages.

**Figure 1 |:**
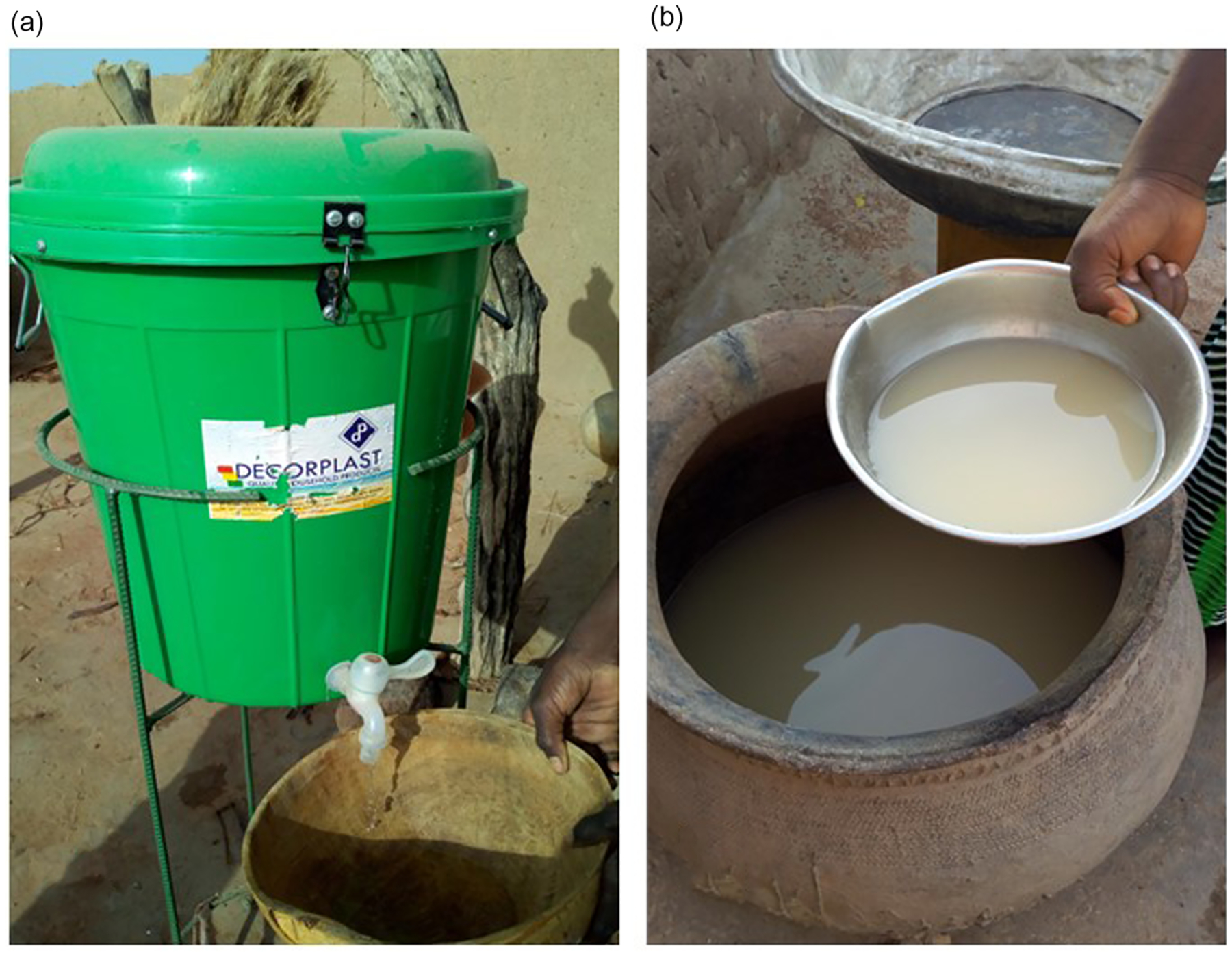
(a) The final design of the safe storage container implemented in intervention villages. (b) A typical example of traditional water storage practices in control villages.

The final SWSCs included a high-flow rate tap, a latched lid, and an interior plastic guard to prevent the insertion of hands, cups, or other potentially contaminated objects. Containers were fitted into metal stands to elevate them off the ground and improve stability (Figure 1). Containers were distributed with verbal instructions on SWSC cleaning and maintenance, and households were encouraged to fill SWSCs only from ‘safe’ water sources, with examples of safe sources described as boreholes or piped supplies. Implementers conducted a demonstration of filling the SWSC and dispensing water using the tap. Households then practiced fetching water, filling SWSCs, and using the tap with the implementer. Supplementary Material, File SI-4 provides an additional description of the intervention and adaptation process.

### Post-implementation survey procedures

We assessed SWSC presence and use through household surveys. To assess presence, enumerators observed whether a container that met safe storage criteria (i.e., possessed a lid, tap, narrow opening [≤15 cm], and was elevated at least 1 m off the ground) was present in the home. To assess use, enumerators asked households for a sample of water and photographed the storage container from which samples were provided. A reviewer blinded to allocation status verified SWSC use based on whether the containers photographed met safe storage criteria. Household surveys were administered to female heads of household using a mobile data collection application (mWater, New York, NY). Household characteristics included water storage and collection practices, hygiene and sanitation practices, and socioeconomic and demographic indicators. The survey questionnaire is included in Supplementary Material, File SI-5.

### Water quality testing

To quantify HSW quality, female heads of household were asked to provide a glass of water as they normally would serve for drinking. Samples were transferred to sterile 100-mL Whirl-Pak Thiobags (Nasco, Fort Atkinson, WI, USA), and Aquagenx test buds containing defined culture media for the selective growth and enumeration of *E. coli* (Aquagenx LLC, Chapel Hill, NC) were added. Samples were agitated and stored on ice for 15 min before transferring to Aquagenx compartment bag test (CBT) bags. CBTs were incubated at ambient temperature for 24 h. The CBT method produces accurate results with thermal incubation at 35 °C or with incubation at ambient temperatures ranging from 25 to 44.5 °C (Stauber *et al*. 2014). Ambient temperatures ranged from 30 to 35 °C during the study period. Lower and upper detection limits are <1 and ≥100 colony forming units (CFUs) per 100 mL, respectively.

For source water samples, members of the village WaSH committee were asked to identify all water sources used in the community at both baseline and end line. Enumerators visited each source to collect a sample using sterile Whirl-Pak^®^ Thiobags. Subsequent procedures for the addition of growth media and incubation were as described above.

Following incubation, enumerators read the CBT results and converted these to the most probable number (MPN) of CFUs per 100 mL sample using the manufacturer’s published tables (Aquagenx 2013). Enumerators also photographed CBTs using the mobile survey platform. Household CBT photographs were verified by an independent reviewer who was blinded to allocation status.

We matched households to nearby water sources based on source type and proximity, as determined using global positioning satellite (GPS) coordinates. During household surveys, participants indicated the source type used for their most recent water collection (e.g., borehole and surface water). For each household, we calculated the average MPN of all sources within a 100-m radius matching the source type households last used when collecting water (hereafter ‘last-used water sources’).

### Rainfall data

Rainfall is associated with water quality (Kistemann *et al*. 2002). Baseline and end-line data were spread over several months and spanned both the rainy and dry seasons, so we adjusted for rainfall as a potential confounder. We extracted rainfall data from the CPC Global Unified Gauge-Based Analysis of Daily Precipitation data sets (National Center for Atmospheric Research Staff 2018), which contain daily precipitation for 0.5° longitude by 0.5° longitude grids. We matched rainfall data for the previous day based on the date of sample collection and household GPS coordinates.

### Statistical analysis

We cleaned and analyzed data using *R* version 3.5.0 (Vienna, Austria). We assessed intervention impact using a difference-in-difference model to examine changes in microbial water contamination from baseline to end line in intervention versus control households. To account for the fact that CBTs yield discrete MPN values that approximate count data, we applied two different hierarchical modeling strategies and compared results across each.

First, to model MPN as an approximated bacterial count, we used a hierarchical quasi-Poisson model to account for over-dispersion in bacterial counts (Agresti 2002). Second, we used hierarchical ordinal logistic regression to account for the fact that the CBT produces ordinal values for MPN rather than true counts. We grouped MPN values into log categories (<1, 1–10, 11–100, and >100 per 100 mL), corresponding with microbial risk categories of the WHO that describe conformity with water quality guidelines and low, intermediate, and high risk, respectively (WHO 2011). Ordinal logistic regression calculates the odds ratio of being in the next ordinal category, in this case the odds ratio of microbial contamination in one category versus the next higher log category. All models were run as three-level models with mixed effects, adjusting for clustering at the household and village levels. All baseline and end-line results with analyzable samples were included in the models to reduce bias from loss to follow up (Diggle *et al*. 2002).

The intervention effect was assessed using random intercepts for allocation (intervention vs. control), time, and a time × allocation interaction effect. We adjusted for potential confounding by point-of-use water treatment, soap presence, open defecation, rainfall, and source water quality as fixed effects.

We conducted both intention-to-treat and per-protocol analyses. Assignment for per-protocol analysis was based on reviewer-verified photographs of SWSC use. For pre-protocol analysis, we considered households to have received the intervention if the photograph showing the container used to provide the water sample was verified by the reviewer to the SWSC.

### Ethics

Ethical approval for this study was obtained from the Institutional Review Board of the University of North Carolina-Chapel Hill, USA (No. IRB #15-1607) and the Ethics Committee of the Ministry of Health in Burkina Faso (No. 2015-9-117). We obtained verbal informed consent from all study participants.

## RESULTS

### Baseline characteristics

**Figure 2 |:**
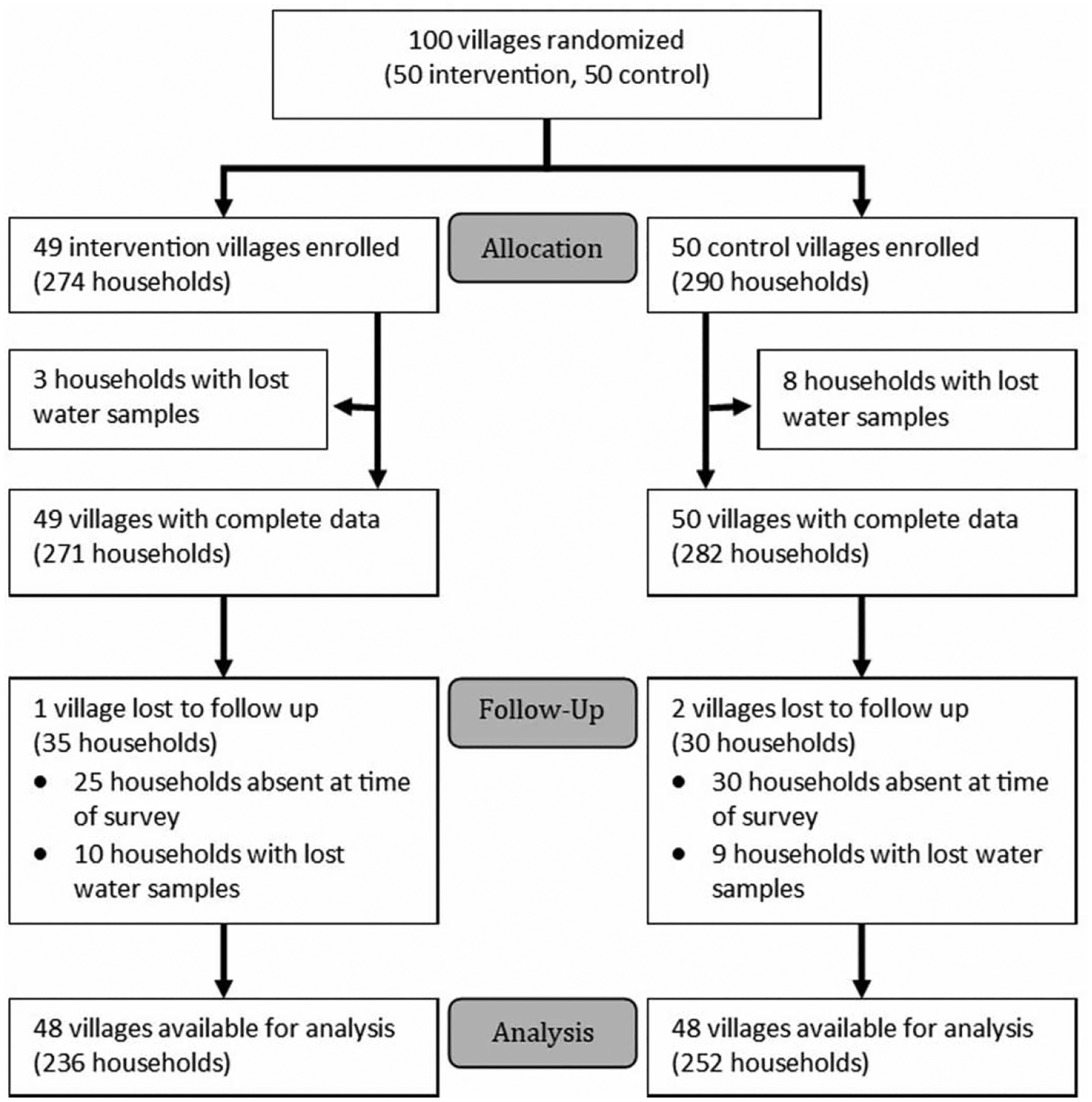
Flow diagram of study participants.

A total of 99 villages (50 control and 49 intervention) and 564 households (290 control and 274 intervention) were enrolled. We initially allocated a total of 600 households (300 per arm). However, a political coup occurred in Burkina Faso shortly after baseline data collection began, causing all study activities to cease. Once data collection resumed, the rainy season had begun, and some areas were unreachable due to flooding and poor road quality. Of enrolled households at baseline, 11 (2%) had water quality samples that were lost due to missing or unmatched barcodes. The final end-line sample comprised 48 control villages (252 households) and 48 intervention villages (236 households) (Figure 2). Two villages were lost to follow up due to mismatched barcodes in a batch of water quality samples, and one village was lost to follow up because all households were absent at the time of survey. For pre-protocol analysis, we analyzed 222 households verified to have received the intervention based on photographs of containers used to provide water samples, and 265 that did not. One household could not be assigned to either group due to a missing photo.

Table 1 displays baseline characteristics of enrolled households. Baseline levels of HSW water contamination and safe water storage practices were similar across intervention and control, as were socioeconomic indicators. Control households were more likely to have used an improved source at their last water collection, and the average MPN for last-used water sources was higher among intervention households (Table 1).

### Intervention uptake and safe water practices following adaptation

Monitoring during adaptation included 96 households that received the initial intervention. Of these, 92.7% self-reported using the SWSC, all of whom used the SWSC tap when asked to demonstrate taking a glass of water to drink. However, enumerators estimated that only 67.3% of households were using their SWSCs based on signs of use (water in the SWSC, stored in accessible location, not used for storing other items). Of water samples taken from in-use SWSCs, 37% (*n* = 26) had no detectable *E. coli*, 29% (*n* = 20) had fewer than 10 MPN/100 mL sample, 17% (*n* = 12) had fewer than 100 MPN, and 17% (*n* = 12) were at the upper detection limit of 100 MPN.

### End-line intervention uptake and safe water storage practices

At end line, 88.9% of intervention and 5.4% of control households were observed to provide water from an SWSC, indicating some contamination between the trial arms. Despite the presence of guards to prevent the insertion of hands or objects into the SWSC, only 45% of users dispensed water through the tap (47% intervention and 14% control). The remainder used hands, ladles, cups, or other objects to scoop water.

The use of improved water sources increased from baseline to end line to 85.1% among control (+6.0%) and 95.9% among intervention (+32.8%) households. While significantly more control households used improved sources at baseline, significantly more intervention households used improved sources at end line (percentage difference: 10.8% [95% CI 5.5, 16.3]). Changes in improved source use corresponded with changes in the microbial contamination of last-used water sources (control: 25.7 MPN/100 mL; intervention: 17.9; and difference: 7.8 [95% CI 3.1, 12.4]). Intervention uptake and safe water storage practices did not differ meaningfully across regions.

### HSW quality

Baseline contamination levels of HSW were comparable across control and intervention households, with mean MPN of *E. coli* per 100 mL samples of 49.8 and 50.9, respectively. The distribution of samples by log MPN categories was also comparable at baseline (Table 2). End-line contamination was significantly lower in intervention households for both intention-to-treat (difference: 19.2 MPN, [95% CI 11.1, 27.1]) and per-protocol analyses (difference: 25.6 MPN [95% CI: 17.6, 33.4]).

Intention-to-treat analysis indicated a −0.42 log (95% CI −0.46, −0.38) reduction in MPN attributable to the intervention in unadjusted quasi-Poisson models. The intervention effect remained significant in models adjusting for source water quality (−0.29 log [95% CI −0.22, −0.26]) or for all other confounders except source quality (−0.39 log [95% CI −0.43, −0.35]). Models adjusted for source water and all other confounders together indicated a non-significant reduction attributable to the intervention. Ordinal logistic models were similar, except that all unadjusted and adjusted models indicated a significant protective intervention effect, with odds ratios of being in the next higher contamination risk level ranged from 0.36 (unadjusted, 95% CI 0.22, 0.57) to 0.50 (fully adjusted, 95% CI 0.22, 0.57) (Table 3).

Per-protocol analysis indicated a −0.56 log (95% CI −0.60, 0.52) reduction in MPN attributable to the intervention in unadjusted models. All adjusted models demonstrated a significant intervention effect, ranging from a −0.55 log reduction (95% CI −0.59, −0.51) in models adjusted for source water quality only to −0.44 (95% CI −0.48, −0.40) after adjusting for all confounders including source quality. Per-protocol ordinal logistic models similarly demonstrated a significant protective effect for all models, with odds ratios ranging from 0.24 (unadjusted, 95% CI 0.15, 0.39) to 0.31 (fully adjusted, 95% CI 0.19, 0.51) (Table 3).

## DISCUSSION

We conducted a cluster-randomized trial of an SWSC intervention adapted to the context of rural Burkina Faso. The intervention achieved a −0.42 log reduction in *E. coli* contamination levels in intention-to-treat analysis using unadjusted quasi-Poisson models. Adjusting for all confounders indicated a non-significant log reduction of −0.23. We also modeled MPN outcomes as ordinal categories corresponding to WHO risk levels for microbial water safety. Ordinal logistic models demonstrated consistently and significantly reduced odds of being in a higher contamination level for all models. Non-significant effects of fully adjusted quasi-Poisson models may be observed in part because our sample size was powered to detect significant differences in the presence versus absence of *E. coli* rather than incremental reductions in a contamination level.

Source water quality is a critical moderator of safe storage interventions. Safe storage protects water from contamination during transport and storage, but reduces contamination levels relative to that of the source. When SWSCs were distributed, intervention households received messaging promoting improved source use, while control households did not. While improved source use increased from baseline to end line among both control and intervention arms, increases were substantially greater among intervention households (+32.8% for intervention households vs. +6.0% for control households).

In the analysis presented here, we adjust for source water quality by calculating the average MPN of all sources matching the last type used within a 100-m radius. We were unable to match households to the specific source last used, and as a proxy assumed that households use sources in close proximity that matched the source type reported by respondents. We assessed the sensitivity of this assumption by also varying the radius to 200, 500, and 1,000 m and by adjusting with a categorical dummy variable for the type of source last used (e.g., borehole and standpipe) and as a binary unimproved/improved variable. Results across models were broadly similar. Results can be viewed in Supplementary Material, File SI-6. We did not adjust our analyses for socioeconomic indicators. Socioeconomic indicators were balanced across intervention and control groups at baseline (Table 1) and were not meaningfully associated with container use or other outcome variables. As such, we did not adjust for these indicators in order to achieve more parsimonious models (Jager *et al*. 2008).

Most models adjusting for source water quality maintained a smaller but still protective effect of safe storage, though confidence intervals were wider. This suggests that both SWSC use and increases in improved water source use contributed to water quality improvements. This finding underscores the importance of delivering SWSC interventions in combination with water source improvements or in areas where sources are known to be safe.

Providing behavior change messaging to promote safe source use is also an important component of SWSC interventions. The fact that intervention households were able to achieve and sustain high improved source use throughout the trial period suggests that the distribution of SWSCs may be an effective trigger for facilitating behavior change around water source selection and use. This may be particularly relevant in contexts characterized by households using portfolios of multiple sources for different domestic needs. However, we found substantial that proportions of households did not correctly dispense water through the SWSC tap (55% inserted scoops, cups, hands, or other items to dispense water). This suggests that additional PDSA cycles to adapt and improve behavior change messages to ensure that correct use is achieved and sustained over time may further improve SWSC interventions.

Our results also align with findings from other trials that report significant water quality improvements from safe storage. Trials in Benin (Günther & Schipper 2013) and Malawi (Roberts *et al*. 2001) each found approximately 70% lower *E. coli* contamination among safe storage users relative to control. Compared to intervention households in the Ghana pilot, a higher percentage of Burkinabe intervention households had detectable *E. coli* in HSW samples (71.6 vs. 59.8%), despite higher uptake of safe storage (88.9 vs. 57%).

Bias in self-reported SWSC usage may partially account for differences in container usage. Usage rates monitored during adaptation were substantially higher for self-reported versus observed signs of use (92.7 vs. 67.3%). Previous studies have consistently shown that the use of safe storage and other point-of-use water quality interventions decreases over time (Clasen *et al*. 2015). We similarly observed decreases in self-reported use from baseline to end line (92.7–88.9%), though more detailed enumerator observations of use were not included in the end-line survey.

Reactivity is less relevant to microbial outcomes, as outcomes that are not self-reported are less subject to conscious or subconscious bias by participants or evaluators (Clasen & Boisson 2016). Furthermore, water quality samples were assigned a numbered barcode upon collection. Following incubation, test results were linked with the same barcode. Allocation was not recorded on CBTs, reducing the likelihood of bias. Other potential explanations for differences in an intervention effect across settings include the length of follow-up (2.3 years Burkina Faso, 2.5 years Ghana) intervention components in Ghana to improve water source functionality that was not included in the Burkinabe intervention and contextual factors such as household WaSH practices, geographic, and seasonal differences in rainfall and flooding, which are known to impact water quality (Wright *et al*. 2004; Kostyla *et al*. 2015). Finally, source type distributions were different, with dug wells more prevalent in the Burkinabe context.

We collected data on water quality using CBTs, which measure *E. coli* concentration. CBTs are suitable for low-resource settings where access to laboratory infrastructure is limited, and CBTs produce comparable results to other techniques such as ultra-membrane filtration (Stauber *et al*. 2014). However, CBTs do not measure other important indicators of water quality, such as fecal coliforms and fecal *Streptococci*. In this trial, we selected CBTs over other tests that include a broader array of indicator species to prioritize ease of use throughout multiple, rapid rounds of testing and iteration for adaptation. This decision also reflects the fact that water quality was only one factor in our adaptation decision matrix, and adaptation also considered adoption, feasibility, and cost of SWSCs. However, future studies may wish to test for a broader array of indicator species to more holistically evaluate water quality.

To our knowledge, this trial is the first to explore PDSA as a possible technique to adapt water quality interventions. SWSCs were originally designed in Ghana and adapted to Burkina Faso in this trial using systematic, data-driven PDSA cycles. Systematic adaptations help to ensure that core functions of an intervention are preserved while improving contextual fit (Kirk *et al*. 2020). In this case, we assessed microbial water quality to ensure that the SWSCs’ ability to protect source water quality was preserved while adapting aspects of the container design to improve feasibility, acceptability, and cost.

PDSA cycles were originally developed in factories in the manufacturing sector, where improvement needs and results could often be readily observed in real-time. In contrast, households receiving rural WaSH interventions are typically dispersed, remote, and/or difficult to access due to factors such as poor road quality and seasonal flooding. These challenges make ongoing monitoring, evaluation, and improvement logistically difficult, time-consuming, and expensive (Fisher *et al*. 2020). Local artisans, supply manufacturers and importers, and WaSH implementing agencies may be geographically distant from recipient households, as they were in both here and the Ghana trial, making timely translation of evidence into action challenging.

Despite these challenges, this trial demonstrates that data-driven systematic adaptations using PDSA are feasible in rural WaSH, provided that adequate funding and support are available. PDSA is a process designed to improve contextual fit, effectiveness, or both. We applied PDSA to adapt an SWSC intervention based on local needs and priorities. However, the methods used in this study for intervention adaptation could be similarly applied for adapting other WaSH products, such as adapting toilets to retain functionality to safely isolate feces while improving acceptability to users, affordability, manufacturing feasibility, and sustainability. We did not apply PDSA cycles to adapt the messaging around safe source use delivered in this study, but we suggest that PDSA may be a useful tool to adapt behavior change messaging as well. Future studies applying PDSA to adapt different interventions in a wider variety of contexts would be useful to explore its applicability as a tool to improve WaSH intervention delivery.

Given the proof of concept that PDSA methods are feasible, future research using head-to-head trials comparing interventions developed and adapted using different approaches (e.g., CQI-based vs. other implementation support approaches, rather than a no-intervention control) would help build the understanding of how best to improve the implementation of water quality and other WaSH interventions for which effectiveness under high adoption and sustainability conditions is already well established, and achieving these conditions is the primary challenge. For such interventions where efficacy under high sustained use has already been demonstrated, using implementation outcomes such as adoption and sustainability as the primary trial outcome would reduce the sample size needed to achieve adequate statistical power, compared to the sample size needed to detect small differences in microbial or health outcomes between groups (Brown *et al*. 2017).

## CONCLUSIONS

We assessed the impact of an SWSC intervention on HSW in rural Burkina Faso. We adapted the SWSC to local user preferences and manufacturer capabilities using PDSA cycles. The SWSC intervention achieved significant reductions in *E. coli* contamination of HSW and had high levels of sustained use among intervention households. These results suggest that PDSA cycles are a viable tool to adapt WaSH technologies to improve contextual fit while preserving their core functions that protect against exposure to fecal pathogens.

## Supplementary Material

SI file 2

SI file 5

SI file 6

SI file 3

SI file 4

SI file 1

## Figures and Tables

**Table 1 |:** Baseline demographic and WaSH characteristics of included households

			Difference
	Control 50 villages 290 households	Intervention 49 villages 274 households	Intervention vs. control (95% confidence interval)
**WaSH characteristics**			
Water storage container met all criteria for safe storage	0.0%	0.4%	+ 0.4% (−0.4, 1.4)
Container has lid	65.2%	61.7%	−3.5% (−11.8, 4.8)
Container has narrow opening	14.8%	10.9%	−3.9% (−9.7, 2.0)
Container has tap	0%	0.7%	+ 0.7% (−0.6, 2.1)
Point-of-use water treatment practiced	1.0%	2.9%	+ 1.9% (−0.8, 4.5)
Last water source used was improved	75.5%	63.1%	−12.4% (−20.3, −4.5)
*E. coli* contamination of all water sources matching the last type used by the household within 100 m, MPN (SD)^a^	23.8 (30.6)	33.3 (34.5)	+ 9.5 (4.0, 14.9)
Household has soap available	35.5%	34.7%	−0.8% (−9.1, 7.4)
Any household member practices open defecation	51.0%	46.0%	−5.0% (−13.6, 3.5)
**Demographic and socioeconomic characteristics**			
Region, *n* (%)			n/a
Boucle du Mouhoun	99 (34.1)	72 (26.3)	
Centre	18 (6.2)	36 (13.1)	
Centre-Est	107 (36.9)	75 (27.4)	
Centre-Ouest	30 (10.3)	59 (21.5)	
Nord	15 (5.2)	20 (7.3)	
Sud-Ouest	21 (7.2)	12 (4.4)	+ 0.2 (−0.1, 0.5)
Household floor made from durable materials^b^	47.9%	47.8%	−0.1% (−8.5, 8.2)
Household walls made from durable materials^c^	23.4%	19.3%	−4.1% (−11.2, 3.0)
Household roof made from durable materials^d^	61.0%	69.7%	+ 8.7% (5, 16.8)
Household has electricity	20.3%	20.8%	+ 0.5% (−6.6, 7.5)
Primary occupation of main income earner			n/a
Farming or other agricultural work	80.7%	82.8%	
Food preparation	1.7%	0.4%	
Construction or other craft	1.0%	1.1%	
Other	16.6%	14.6%	
No occupation	0.0%	1.1%	

a MPN of *E. coli* CFUs per 100 mL sample.

b Durable flooring materials defined as cement, ceramic, wood, carpet, vinyl, or bamboo.

c Durable wall materials defined as cement, stone, brick, adobe, wood, or plastic.

d Durable roofing materials defined as metal, ceramic, cement, roofing shingles, plastic, or roofing asphalt.

**Table 2 |:** Baseline and end-line household water contamination outcomes

	Baseline		End line – intention-to-treat		End line – per-protocol	
	Control *n* = 282	Intervention *n* = 271	Control *n* = 252	Intervention *n* = 236	Control *n* = 265	Intervention *n* = 222
*E. coli* per 100 mL, MPN (SD)	49.8 (43.9)	50.9 (44.6)	64.9 (43.8)	45.7 (46.1)	67.4 (43.0)	41.8 (45.3)
MPN per 100 mL, *n* (%)						
<1	46 (16.3)	45 (16.6)	32 (12.7)	63 (26.7)	32 (12.1)	63 (28.4)
1 to <10	45 (16.0)	44 (16.2)	25 (9.9)	43 (18.2)	23 (8.7)	44 (19.8)
10 to <100	77 (27.3)	67 (24.7)	46 (18.3)	36 (15.3)	46 (17.4)	36 (16.2)
≥100	114 (40.4)	115 (42.4)	149 (59.1)	94 (39.8)	164 (61.9)	79 (35.6)

The MPN of CFUs per 100 mL sample was assessed using the compartment bag test.

**Table 3 |:** Intervention effect sizes for quasi-Poisson and ordinal logistic regression models with intention-to-treat and per-protocol analysis

	Quasi-Poisson models			
Analysis	Unadjusted	Adjusted – all but source water quality	Adjusted – source water quality only	Fully adjusted
Intention-to-treat	−0.42 (−0.46, −0.38)	−0.39 (−0.43, −0.35)	−0.29 (−0.33, −0.26)	−0.23 (−0.59, 0.13)
Per-protocol	−0.56 (−0.60, −0.52)	−0.55 (−0.59, −0.51)	−0.44 (−0.48, −0.41)	−0.44 (−0.48, −0.40)
	Ordinal logistic models			
Analysis	Unadjusted	Adjusted – all but source water quality	Adjusted – source water quality only	Fully adjusted
Intention-to-treat	−1.03 (−1.50, −0.56)	−0.95 (−1.42, −0.48)	−0.74 (−1.21, −0.26)	−0.69 (−1.17, −0.21)
Per-protocol	−1.42 (−1.91, −0.93)	−1.36 (−1.85, −0.86)	−1.14 (−1.64, −0.64)	−1.11 (−1.61, −0.61)

Effect sizes represent coefficients for time × treatment interaction with 95% confidence intervals indicated in parentheses. Quasi-Poisson model coefficients represent the log reduction in *E. coli* count, and ordinal logistic model coefficients represent the change in the log-odds of moving to the next lowest contamination level (e.g., a shift from 10– < 100 MPN/100 mL to 1– < 10 MPN/100 mL). Models adjusted for all variables except source water quality were adjusted for point-of-use water treatment, soap presence, open defecation, and rainfall. Fully adjusted models were adjusted for all of the above plus source water quality.

## Data Availability

All relevant data are available from an online repository or repositories (https://doi.org/10.15139/S3/IGSF54).

## HIGHLIGHTS

- Safe water storage interventions can improve health, but uptake and sustained use are challenging.
- Adapting storage containers to local preferences and manufacturing abilities improves uptake and sustainability.
- Plan-Do-Study-Act cycles are a tool to adapt interventions.
- Our adapted safe water storage intervention achieved high sustained use and a significant reduction in microbial contamination of stored drinking water.
