# Supplementary material for: Adapting a safe water storage container to improve household stored water quality in rural Burkina Faso: a cluster randomized trial
Source: J Water Sanit Hyg Dev. Author manuscript; Available in PMC 2025 Sep 23. (PMC12453111; doi:10.2166/washdev.2021.065)
Supplement: SI file 2 [file NIHMS2111177-supplement-SI_file_2.pdf]

WaterAid Uptake Survey1

Metadata

Date: 1. Date

Time_hour: 2. Time: Enter Hour

Hint: Enter hour based on 24 clock

Time_min: 3. Time: Enter Minutes

geo_code: 4. GPS coordinates

Hint: Make sure GPS coordinates are accurate within 10 meters

Country: 5. Country

◯ Burkina Faso
◯ Ethiopia
◯ Ghana
◯ India
◯ Mali
◯ Mexico
◯ Niger

Region: 6. Region

◯ Boucle du Mouhoun
◯ Centre
◯ Centre-Est
◯ Centre-Ouest
◯ Est
◯ Nord
◯ Sahel
◯ Sud-Ouest

Province: 7. Province

- ◯ Balé
- ◯ Kadiogo
- ◯ Boulgou
- ◯ Koulpélogo
- ◯ Boulkiémdé
- ◯ Sanguié
- ◯ Tapoa
- ◯ Passoré
- ◯ Séno
- ◯ Ioba

If 7. Province is Balé:
Commune: 8.1 Commune

- ◯ Bagassi
- ◯ Bana
- ◯ Boromo
- ◯ Fara
- ◯ Oury
- ◯ Pa
- ◯ Poura
- ◯ Siby
- ◯ Yaho

If 7. Province is Boulgou:
Commune: 8.2 Commune

- ◯ Garango
- ◯ Komtoèga
- ◯ Niaogho
- ◯ Tenkodogo

If 7. Province is Boulkiémdé:
Commune: 8.3 Commune

- ◯ Imasgho
- ◯ Kindi
- ◯ Koudougou
- ◯ Ramongo

If 7. Province is Ioba:

Commune: 8.4 Commune

- ◯ Dano
- ◯ Dissin

If 7. Province is Kadiogo:

Commune: 8.5 Commune

- ◯ Komki Ipala
- ◯ Pabré
- ◯ Saaba
- ◯ Tanghin-Dassouri

If 7. Province is Koulpélogo:

Commune: 8.6 Commune

- ◯ Comin Yanga
- ◯ Dourtenga
- ◯ Lalgaye
- ◯ Ouargaye
- ◯ Sangha
- ◯ Soudougui
- ◯ Yargatenga
- ◯ Yondé

If 7. Province is Passoré:

Commune: 8.7 Commune

- ◯ Arbollé
- ◯ Bokin
- ◯ Kirsi

If 7. Province is Sanguié:

Commune: 8.8 Commune

- ◯ Dassa
- ◯ Didyr

If 7. Province is Séno:

Commune: 8.9 Commune

- ◯ Dori

If 7. Province is Tapoa:

Commune: 8.10 Commune

- ◯ Diapaga

Org: 9. Organization collecting the data

- ◯ CARE
- ◯ CRS
- ◯ Helvetas
- ◯ Living Water International
- ◯ One Drop
- ◯ UNC
- ◯ UNICEF
- ◯ WaterAid
- ◯ World Vision
- ◯ WSA

Enumerator: 10. Your name

Community_name: 11. Name of Village

Community: 12. Village ID

House_ID: 13. Household ID

Hint: If no ID flag is present, ask the respondent's permission to place an ID flag on the house so you can find it again later.

Consent: 14. Has informed consent been obtained?

Hint: If the respondent declines, thank them and conclude the survey

- ◯ Yes
- ◯ No

If 14. Has informed consent been obtained? is Yes:

Participant_name: 15. Full name of respondent

If 14. Has informed consent been obtained? is Yes:

Household Characteristics

Household_size: 16. How many people live in your household? Household means the number of people living under this roof, including you.

[ ]

Compound: 17. [Direct Observation] Does the respondent live in a multi-household compound?

- ◯ Yes
- ◯ No
- ◯ Not applicable
- ◯ Don't know

If 17. [Direct Observation] Does the respondent live in a multi-household compound? is Yes:

Compound_size: 18. What is the total number of people living in this compound including yourself?

[ ]

If 14. Has informed consent been obtained? is Yes:

Water reliability

Last_source: 136. What water source did you most recently fetch water from?

- ◯ Piped water into dwelling
- ◯ Piped water to yard/plot
- ◯ Public tap/standpipe
- ◯ Borehole
- ◯ Protected dug well
- ◯ Unprotected dug well
- ◯ Protected spring
- ◯ Unprotected spring
- ◯ Rainwater collection
- ◯ Pay another person to fetch/buy filled containers from a vendor
- ◯ Bottled water/sachet water/"pure water"
- ◯ Cart with small tank/drum
- ◯ Tanker truck
- ◯ Surface water (river, dam, lake, pond, stream, canal, irrigation channels)
- ◯ Other (please specify)

[ ]

- ◯ Not applicable
- ◯ Don't know
- ◯ Declined to state

If 14. Has informed consent been obtained? is Yes:

Household water

InterventionHH: Has anyone brought you a container for storing drinking water in the last three months?

- ◯ Yes
- ◯ No
- ◯ Don't know
- ◯ Declined to state

If Has anyone brought you a container for storing drinking water in the last three months? is Yes:
show_container: Can you show me the container?

- ◯ Yes
- ◯ No
- ◯ Don't know
- ◯ Declined to show

If Has anyone brought you a container for storing drinking water in the last three months? is Yes:
container_obs: [Observe] is the WaterAid safe water storage container present in the home?

- ◯ Yes
- ◯ No
- ◯ Don't know

If Has anyone brought you a container for storing drinking water in the last three months? is Yes:
Family_use: Is your family using this container?

- ◯ Yes
- ◯ No
- ◯ Not applicable
- ◯ Don't know
- ◯ Declined to state

If Is your family using this container? is Yes:
Can you show me how you take water from the container to drink?

- ◯ Fetched from tap
- ◯ Pouring
- ◯ Dipping cup or other container
- ◯ Other (please specify)

- ◯ Declined to show

If Is your family using this container? is Yes:

container_purpose: For what purpose is your family using the container?

- ◯ Storing water for drinking
- ◯ Storing water for other purposes
- ◯ Storing things other than water
- ◯ For a stool
- ◯ Other (please specify)

If Is your family using this container? is No:

whynot_use: Why is your family not using the container currently?

- ◯ Tap broke
- ◯ Container reservoir broke
- ◯ Stand broke
- ◯ Lid broke
- ◯ Other part broke
- ◯ Container was lost
- ◯ Container stolen
- ◯ Container borrowed and not returned
- ◯ Container used for another purpose
- ◯ Stand used for another purpose
- ◯ Water in container had bad taste
- ◯ Water in container was too hot
- ◯ Container was too hard to fill
- ◯ Container was too hard to dispense water from
- ◯ Container was too unstable
- ◯ Other (please specify)

- ◯ Don't know
- ◯ Declined to state

If Why is your family not using the container currently? is Other part broke:

part_specify: Which part broke?

If Why is your family not using the container currently? is Container used for another purpose:

purpose_container: What other purpose was the container used for?

If Why is your family not using the container currently? is Stand used for another purpose:

purpose_stand: What other purpose was the stand used for?

If Has anyone brought you a container for storing drinking water in the last three months? is Yes:

adults_drink: Where do adults in this household mainly take water to drink when they are at home?

◯ WaterAid safe water storage container
◯ Other container
◯ Directly from household tap

If Where do adults in this household mainly take water to drink when they are at home? is WaterAid safe water storage container:

adults_other_con: Do adults in this household also drink from other containers when they are at home?

◯ Yes
◯ No
◯ Don't know
◯ Declined to state

If Has anyone brought you a container for storing drinking water in the last three months? is Yes:

child_drink: Where do children over the age of five years old in this household mainly take water to drink when they are at home?

◯ WaterAid safe water storage container
◯ Other container
◯ Directly from household tap

If Where do children over the age of five years old in this household mainly take water to drink when they are at home? is WaterAid safe water storage container:

child_other_con: Do children in this household also drink from other containers when they are at home?

◯ Yes
◯ No
◯ Don't know
◯ Declined to state

If Has anyone brought you a container for storing drinking water in the last three months? is Yes:

lessfive_drink: When children younger than five years old in this household drink water at home, where is this water taken from?

◯ WaterAid safe water storage container
◯ Other container
◯ Directly from household tap
◯ Not applicable
◯ Don't know
◯ Declined to state

If When children younger than five years old in this household drink water at home, where is this water taken from? is WaterAid safe water storage container:

lessfive_other_con: Is water for children under five years old in this household also taken from other containers?

◯ Yes
◯ No
◯ Not applicable
◯ Don't know
◯ Declined to state

If Is your family using this container? is Yes:

time_lastdrink: How long ago was the last time you took water from the safe storage container to drink?

If [Observe] is the WaterAid safe water storage container present in the home? is Yes:

container_water: [Observe] Does the WaterAid safe water storage container have water in it?

◯ Yes
◯ No
◯ Don't know

If [Observe] is the WaterAid safe water storage container present in the home? is Yes:

container_cover: [Observe] Is the WaterAid safe water storage container completely covered?

◯ Yes
◯ No
◯ Don't know

If [Observe] is the WaterAid safe water storage container present in the home? is Yes:

container_ground: [Observe] Is the WaterAid safe water storage container on a stand (not on the ground)?

◯ Yes
◯ No
◯ Don't know

If [Observe] is the WaterAid safe water storage container present in the home? is Yes:

container_cracked: [Observe] Is any part of the safe water storage container cracked or broken?

◯ Yes
◯ No
◯ Don't know

If [Observe] Is any part of the safe water storage container cracked or broken? is Yes:

Part_cracked: [Observe] Which parts of the safe water storage container are cracked or broken? [check all that apply]

☐ Tap
☐ Container reservoir
☐ Stand
☐ Lid
☐ Other (please specify)

☐ Don't know

If [Observe] is the WaterAid safe water storage container present in the home? is Yes:

Container_use: [Observe] Does the safe water storage container show signs of recent use?

◯ Yes
◯ No
◯ Don't know

If [Observe] Does the safe water storage container show signs of recent use? is No:

[Observe] What indications did you observe that this storage container is not being used?(Mark all that apply)

☐ No water in container
☐ Container stored in an inaccessible location
☐ Container used for a different purpose (such as storing grain or other things besides water)
☐ Other objects stacked on top of container
☐ Other (please specify)

☐ Don't Know
☐ Not Applicable

Comments...

Treat: 170. Do you treat your water to make it safer for drinking?

◯ Yes
◯ No
◯ Not applicable
◯ Declined to state
◯ Don't know

If 170. Do you treat your water to make it safer for drinking? is Yes:

Treat_type: 171. What do you usually do to the water to make it safer to drink? Anything else? [Do not read choices, mark all items mentioned]

☐ Boil
☐ Add bleach/chlorine
☐ Strain it through a cloth
☐ Use a water filter (ceramic or sand or composite, etc)
☐ Solar disinfection
☐ Let it stand and settle
☐ Other (please specify)

☐ Don't know
☐ Declined to state

If [Observe] Does the safe water storage container show signs of recent use? is No:

What changes to this container might make people more likely to use it for storing their drinking water?

If 170. Do you treat your water to make it safer for drinking? is Yes:

Treat_frequency: 172. In the last two weeks, have you treated your water:

◯ Everyday
◯ Most of the days
◯ Half of the days
◯ Less than half of the days
◯ Not at all
◯ Don't know
◯ Declined to state

☐ Don't Know

Sample_ID1: 173. Water sample ID

Sample_ID: 174. Water sample ID (confirm)

If 173. Water sample ID is not equal to 888:

Storage_char: 175. Can you serve me some water the way you normally take it? [Direct Observation] Does/is the drinking-water storage container: [Mark all that apply]

☐ Have a lid that is completely covering it
☐ Have a narrow opening
☐ Have a tap or spigot
☐ Beyond reach of animals (1 meter or more from the ground)
☐ Clean (free of dirt- debris- garbage- faecal matter- etc.)
☐ Does not have container
☐ Other (please specify)

☐ Declined to show
☐ None of the above

If 173. Water sample ID is not equal to 888:

Method_serve: 176. [Photo] Take a picture of the respondent taking water from the drinking-water storage container the way they normally take it

If 173. Water sample ID is not equal to 888:

Utensil: 177. [Direct Observation] What was used to take water from the storage container?

- ◯ Nothing (water poured or dispensed through a spigot or spout)
- ◯ Dipper or ladle
- ◯ Bucket
- ◯ Hand
- ◯ Cup or bowl or jar or can
- ◯ Other (please specify)
- ◯ Not applicable

If 173. Water sample ID is not equal to 888:

178. What is the source of this [the water that is sampled] water? [Mark all that apply]

- ☐ Piped water into dwelling
- ☐ Piped water to yard/plot
- ☐ Public tap/standpipe
- ☐ Borehole
- ☐ Protected dug well
- ☐ Unprotected dug well
- ☐ Protected spring
- ☐ Unprotected spring
- ☐ Rainwater collection
- ☐ Pay another person to fetch/buy filled containers from a vendor
- ☐ Bottled water/sachet water/"pure water"
- ☐ Cart with small tank/drum
- ☐ Tanker truck
- ☐ Surface water (river, dam, lake, pond, stream, canal, irrigation channels)
- ☐ Other (please specify)
- ☐ Not applicable
- ☐ Don't know
- ☐ Declined to state

Untitled Section

Notes: 249. Thank the respondent for their time [Record your notes here]

End_hour: 250. End time: hour

Hint: Enter hour based on 24 clock

End_min: 251. End time: minute
