# Supplementary material for: Adapting a safe water storage container to improve household stored water quality in rural Burkina Faso: a cluster randomized trial
Source: J Water Sanit Hyg Dev. Author manuscript; Available in PMC 2025 Sep 23. (PMC12453111; doi:10.2166/washdev.2021.065)
Supplement: SI file 5 [file NIHMS2111177-supplement-SI_file_5.pdf]

WaterAid Baseline Household Survey

Metadata

Date: 1. Date

Time_hour: 2. Time: Enter Hour

Hint: Enter hour based on 24 clock

Time_min: 3. Time: Enter Minutes

geo_code: 4. GPS coordinates

Hint: Make sure GPS coordinates are accurate within 10 meters

Country: 5. Country

- ◯ Burkina Faso
- ◯ Ethiopia
- ◯ Ghana
- ◯ India
- ◯ Mali
- ◯ Mexico
- ◯ Niger

Region: 6. Region

- ◯ Boucle du Mouhoun
- ◯ Centre
- ◯ Centre-Est
- ◯ Centre-Ouest
- ◯ Est
- ◯ Nord
- ◯ Sahel
- ◯ Sud-Ouest

Province: 7. Province

- ◯ Balé
- ◯ Kadiogo
- ◯ Boulgou
- ◯ Koulpélogo
- ◯ Boulkiémdé
- ◯ Sanguié
- ◯ Tapoa
- ◯ Passoré
- ◯ Séno
- ◯ Ioba

If 7. Province is Balé:
Commune: 8.1 Commune

- ◯ Bagassi
- ◯ Bana
- ◯ Boromo
- ◯ Fara
- ◯ Oury
- ◯ Pa
- ◯ Poura
- ◯ Siby
- ◯ Yaho

If 7. Province is Boulgou:
Commune: 8.2 Commune

- ◯ Garango
- ◯ Komtoèga
- ◯ Niaogho
- ◯ Tenkodogo

If 7. Province is Boulkiémdé:
Commune: 8.3 Commune

- ◯ Imasgho
- ◯ Kindi
- ◯ Koudougou
- ◯ Ramongo

If 7. Province is Ioba:
Commune: 8.4 Commune

◯ Dano
◯ Dissin

If 7. Province is Kadiogo:
Commune: 8.5 Commune

◯ Komki Ipala
◯ Pabré
◯ Saaba
◯ Tanghin-Dassouri

If 7. Province is Koulpélogo:
Commune: 8.6 Commune

◯ Comin Yanga
◯ Dourtenga
◯ Lalgaye
◯ Ouargaye
◯ Sangha
◯ Soudougui
◯ Yargatenga
◯ Yondé

If 7. Province is Passoré:
Commune: 8.7 Commune

◯ Arbollé
◯ Bokin
◯ Kirsi

If 7. Province is Sanguié:
Commune: 8.8 Commune

◯ Dassa
◯ Didyr

If 7. Province is Séno:
Commune: 8.9 Commune

◯ Dori

If 7. Province is Tapoa:
Commune: 8.10 Commune

◯ Diapaga

Org: 9. Organization collecting the data

◯ CARE
◯ CRS
◯ Helvetas
◯ Living Water International
◯ One Drop
◯ UNC
◯ UNICEF
◯ WaterAid
◯ World Vision
◯ WSA

Enumerator: 10. Your name

Community_name: 11. Name of Village

Community: 12. Village ID

House_ID: 13. Household ID

Hint: If no ID flag is present, ask the respondent's permission to place an ID flag on the house so you can find it again later.

Consent: 14. Has informed consent been obtained?

Hint: If the respondent declines, thank them and conclude the survey

◯ Yes
◯ No

If 14. Has informed consent been obtained? is Yes:

Participant_name: 15. Full name of respondent

If 14. Has informed consent been obtained? is Yes:

Household Characteristics

Household_size: 16. How many people live in your household? Household means the number of people living under this roof, including you.

Compound: 17. [Direct Observation] Does the respondent live in a multi-household compound?

- ◯ Yes
- ◯ No
- ◯ Not applicable
- ◯ Don't know

If 17. [Direct Observation] Does the respondent live in a multi-household compound? is Yes:

Compound_size: 18. What is the total number of people living in this compound including yourself?

Children: 19. How many children under the age of 5 live in your household?

If 19. How many children under the age of 5 live in your household? is greater than 0:

Diarrhea: 20. Has one or more of these children under the age of 5 had diarrhea in the past two weeks? Diarrhea means having three or more loose or liquid stools within 24 hours.

- ◯ Yes
- ◯ No
- ◯ Not applicable
- ◯ Don't know
- ◯ Declined to state

Children_school: 21. Are there any children in your house who are attending primary or secondary school?

- ◯ Yes
- ◯ No
- ◯ Not applicable
- ◯ Don't know
- ◯ Declined to state

If 21. Are there any children in your house who are attending primary or secondary school? is Yes:

Missed_school: 22. Has one or more of these children who attend school missed one or more days of school in the past two weeks due to illness?

- ◯ Yes
- ◯ No
- ◯ Not applicable
- ◯ Don't know
- ◯ Declined to state

Education: 23. What is the highest level of school you have completed?

- ◯ Never attended school
- ◯ Some primary school (did not complete)
- ◯ Primary (Up to Grade 6)
- ◯ Secondary (Up to Grade 12)
- ◯ University (Above Grade 12)
- ◯ Technical Institute (Above Grade 12)
- ◯ Non-formal education
- ◯ Other (please specify)

[ ]

- ◯ Don't know
- ◯ Declined to state

Participant

Participant_gender: 24. [Direct Observation] Is the participant male or female?

- ◯ Male
- ◯ Female

Participant_age: 25. How old are you?

[ ]

Participant_water: 26. Did you go fetch water yesterday?

- ◯ Yes
- ◯ No
- ◯ Not applicable
- ◯ Don't know
- ◯ Declined to state

If 26. Did you go fetch water yesterday? is Yes:

Participant_trips: 27. How many times did you go to fetch water yesterday?

If 26. Did you go fetch water yesterday? is Yes:

Participant_containers: 28. When you went to fetch water yesterday, which container did you use? [Estimate container size in Liters]

If 26. Did you go fetch water yesterday? is Yes:

Participant_containers_number: 29. Each time you went to fetch water, how many containers like this did you carry and fill?

Person 1

Person1_name: 30. Who else lives in this household? [Record first name only; ask respondent to begin with any other adults in the household, then list all children from oldest to youngest]

Person1_gender: 31. Is [NAME] male or female?

- ◯ Male
- ◯ Female
- ◯ Not applicable
- ◯ Declined to state

Person1_age: 32. How old is [NAME]?

Person1_water: 33. Did [NAME] go to fetch water yesterday?

- ◯ Yes
- ◯ No
- ◯ Not applicable
- ◯ Don't know
- ◯ Declined to state

If 33. Did [NAME] go to fetch water yesterday? is Yes:

Person1_trips: 34. How many times did [NAME] go to fetch water yesterday?

If 33. Did [NAME] go to fetch water yesterday? is Yes:

Person1_containers: 35. When [NAME] went to fetch water, which container did [NAME] use? [Estimate container size in Liters]

If 33. Did [NAME] go to fetch water yesterday? is Yes:

Person1_containers_number: 36. Each time [NAME] goes to fetch water, how many containers like this does [NAME] carry and fill?

Person2: 37. Is there another person to add?

- ◯ Yes
- ◯ No
- ◯ Don't know
- ◯ Declined to state

If 37. Is there another person to add? is Yes:

Person 2

If 37. Is there another person to add? is Yes:

Person2_name: 38. Who else lives in this household? [Record first name only; ask respondent to begin with any other adults in the household, then list all children from oldest to youngest]

If 37. Is there another person to add? is Yes:

Person2_gender: 39. Is [NAME] male or female?

- ◯ Male
- ◯ Female
- ◯ Declined to state

If 37. Is there another person to add? is Yes:

Person2_age: 40. How old is [NAME]?

If 37. Is there another person to add? is Yes:

Person2_water: 41. Did [NAME] go to fetch water yesterday?

◯ Yes
◯ No
◯ Not applicable
◯ Don't know
◯ Declined to state

If 41. Did [NAME] go to fetch water yesterday? is Yes:

Person2_trips: 42. How many times did [NAME] go to fetch water yesterday?

If 41. Did [NAME] go to fetch water yesterday? is Yes:

Person2_containers: 43. When [NAME] went to fetch water, which container did [NAME] use? [Estimate container size in Liters]

If 41. Did [NAME] go to fetch water yesterday? is Yes:

Person2_containers_number: 44. Each time [NAME] goes to fetch water, how many containers like this does [NAME] carry and fill?

If 37. Is there another person to add? is Yes:

Person3: 45. Is there another person to add?

◯ Yes
◯ No
◯ Don't know
◯ Declined to state

If 45. Is there another person to add? is Yes:

Person 3

Person3_name: 46. Who else lives in this household? [Record first name only; ask respondent to begin with any other adults in the household, then list all children from oldest to youngest]

If 45. Is there another person to add? is Yes:

Person3_gender: 47. Is [NAME] male or female?

◯ Male
◯ Female
◯ Declined to state

If 45. Is there another person to add? is Yes:

Person3_age: 48. How old is [NAME]?

If 45. Is there another person to add? is Yes:

Person3_water: 49. Did [NAME] go to fetch water yesterday?

◯ Yes
◯ No
◯ Not applicable
◯ Don't know
◯ Declined to state

If 49. Did [NAME] go to fetch water yesterday? is Yes:

Person3_trips: 50. How many times did [NAME] go to fetch water yesterday?

If 49. Did [NAME] go to fetch water yesterday? is Yes:

Person3_container: 51. When [NAME] went to fetch water, which container did [NAME] use? [Estimate container size in Liters]

If 49. Did [NAME] go to fetch water yesterday? is Yes:

Person3_containers_number: 52. Each time [NAME] goes to fetch water, how many containers like this does [NAME] carry and fill?

If 45. Is there another person to add? is Yes:

Person4: 53. Is there another person to add?

◯ Yes
◯ No
◯ Don't know
◯ Declined to state

If 53. Is there another person to add? is Yes:

Person 4

Person4_name: 54. Who else lives in this household? [Record first name only; ask respondent to begin with any other adults in the household, then list all children from oldest to youngest]

Person4_gender: 55. Is [NAME] male or female?

- ◯ Male
- ◯ Female
- ◯ Declined to state

Person4_age: 56. How old is [NAME]?

If 53. Is there another person to add? is Yes:

Person4_water: 57. Did [NAME] go to fetch water yesterday?

- ◯ Yes
- ◯ No
- ◯ Not applicable
- ◯ Don't know
- ◯ Declined to state

If 57. Did [NAME] go to fetch water yesterday? is Yes:

Person4_trips: 58. How many times did [NAME] go to fetch water yesterday?

If 57. Did [NAME] go to fetch water yesterday? is Yes:

Person4_containers: 59. When [NAME] went to fetch water, which container did [NAME] use? [Estimate container size in Liters]

If 57. Did [NAME] go to fetch water yesterday? is Yes:

Person4_containers_number: 60. Each time [NAME] goes to fetch water, how many containers like this does [NAME] carry and fill?

If 53. Is there another person to add? is Yes:

Person5: 61. Is there another person to add?

◯ Yes
◯ No
◯ Don't know
◯ Declined to state

If 61. Is there another person to add? is Yes:

Person 5

If 61. Is there another person to add? is Yes:

Person5_name: 62. Who else lives in this household? [Record first name only; ask respondent to begin with any other adults in the household, then list all children from oldest to youngest]

If 61. Is there another person to add? is Yes:

Person5_gender: 63. Is [NAME] male or female?

◯ Male
◯ Female
◯ Declined to state

If 61. Is there another person to add? is Yes:

Person5_age: 64. How old is [NAME]?

If 61. Is there another person to add? is Yes:

Person5_water: 65. Did [NAME] go to fetch water yesterday?

◯ Yes
◯ No
◯ Not applicable
◯ Don't know
◯ Declined to state

If 65. Did [NAME] go to fetch water yesterday? is Yes:

Person5_trips: 66. How many times did [NAME] go to fetch water yesterday?

If 65. Did [NAME] go to fetch water yesterday? is Yes:

Person5_containers: 67. When [NAME] went to fetch water, which container did [NAME] use? [Estimate container size in Liters]

If 65. Did [NAME] go to fetch water yesterday? is Yes:

Person5_containers_number: 68. Each time [NAME] goes to fetch water, how many containers like this does [NAME] carry and fill?

If 61. Is there another person to add? is Yes:

Person6: 69. Is there another person to add?

- ◯ Yes
- ◯ No
- ◯ Don't know
- ◯ Declined to state

If 69. Is there another person to add? is Yes:

Person 6

If 69. Is there another person to add? is Yes:

Person6_name: 70. Who else lives in this household? [Record first name only; ask respondent to begin with any other adults in the household, then list all children from oldest to youngest]

If 69. Is there another person to add? is Yes:

Person6_gender: 71. Is [NAME] male or female?

- ◯ Male
- ◯ Female
- ◯ Declined to state

If 69. Is there another person to add? is Yes:

Person6_age: 72. How old is [NAME]?

If 69. Is there another person to add? is Yes:

Person6_water: 73. Did [NAME] go to fetch water yesterday?

- ◯ Yes
- ◯ No
- ◯ Not applicable
- ◯ Don't know
- ◯ Declined to state

If 73. Did [NAME] go to fetch water yesterday? is Yes:

Person6_trips: 74. How many times did [NAME] go to fetch water yesterday?

If 73. Did [NAME] go to fetch water yesterday? is Yes:

Person6_containers: 75. When [NAME] went to fetch water, which container did [NAME] use? [Estimate container size in Liters]

If 73. Did [NAME] go to fetch water yesterday? is Yes:

Person6_containers_number: 76. Each time [NAME] goes to fetch water, how many containers like this does [NAME] carry and fill?

If 69. Is there another person to add? is Yes:

Person7: 77. Is there another person to add?

- ◯ Yes
- ◯ No
- ◯ Declined to state
- ◯ Don't know

If 77. Is there another person to add? is Yes:

Person 7

If 77. Is there another person to add? is Yes:

Person7_name: 78. Who else lives in this household? [Record first name only; ask respondent to begin with any other adults in the household, then list all children from oldest to youngest]

If 77. Is there another person to add? is Yes:

Person7_gender: 79. Is [NAME] male or female?

- ◯ Male
- ◯ Female
- ◯ Declined to state

If 77. Is there another person to add? is Yes:

Person7_age: 80. How old is [NAME]?

If 77. Is there another person to add? is Yes:

Person7_water: 81. Did [NAME] go to fetch water yesterday?

- ◯ Yes
- ◯ No
- ◯ Not applicable
- ◯ Don't know
- ◯ Declined to state

If 81. Did [NAME] go to fetch water yesterday? is Yes:

Person7_trips: 82. How many times did [NAME] go to fetch water yesterday?

If 81. Did [NAME] go to fetch water yesterday? is Yes:

Person7_containers: 83. When [NAME] went to fetch water, which container did [NAME] use? [Estimate container size in Liters]

If 81. Did [NAME] go to fetch water yesterday? is Yes:

Person7_containers_number: 84. Each time [NAME] goes to fetch water, how many containers like this does [NAME] carry and fill?

If 77. Is there another person to add? is Yes:

Person8: 85. Is there another person to add?

- ◯ Yes
- ◯ No
- ◯ Don't know
- ◯ Declined to state

If 85. Is there another person to add? is Yes:

Person 8

If 85. Is there another person to add? is Yes:

Person8_name: 86. Who else lives in this household? [Record first name only; ask respondent to begin with any other adults in the household, then list all children from oldest to youngest]

If 85. Is there another person to add? is Yes:

Person8_gender: 87. Is [NAME] male or female?

- ◯ Male
- ◯ Female
- ◯ Declined to state

If 85. Is there another person to add? is Yes:

Person8_age: 88. How old is [NAME]?

If 85. Is there another person to add? is Yes:

Person8_water: 89. Did [NAME] go to fetch water yesterday?

- ◯ Yes
- ◯ No
- ◯ Not applicable
- ◯ Don't know
- ◯ Declined to state

If 89. Did [NAME] go to fetch water yesterday? is Yes:

Person8_trips: 90. How many times did [NAME] go to fetch water yesterday?

If 89. Did [NAME] go to fetch water yesterday? is Yes:

Person8_containers: 91. When [NAME] went to fetch water, which container did [NAME] use? [Estimate container size in Liters]

If 89. Did [NAME] go to fetch water yesterday? is Yes:

Person8_containers_number: 92. Each time [NAME] goes to fetch water, how many containers like this does [NAME] carry and fill?

If 85. Is there another person to add? is Yes:

Person9: 93. Is there another person to add?

◯ Yes
◯ No
◯ Don't know
◯ Declined to state

If 93. Is there another person to add? is Yes:

Person 9

If 93. Is there another person to add? is Yes:

Person9_name: 94. Who else lives in this household? [Record first name only; ask respondent to begin with any other adults in the household, then list all children from oldest to youngest]

If 93. Is there another person to add? is Yes:

Person9_gender: 95. Is [NAME] male or female?

◯ Male
◯ Female
◯ Declined to state

If 93. Is there another person to add? is Yes:

Person9_age: 96. How old is [NAME]?

If 93. Is there another person to add? is Yes:

Person9_water: 97. Did [NAME] go to fetch water yesterday?

◯ Yes
◯ No
◯ Not applicable
◯ Declined to state
◯ Declined to state

If 97. Did [NAME] go to fetch water yesterday? is Yes:

Person9_trips: 98. How many times did [NAME] go to fetch water yesterday?

If 97. Did [NAME] go to fetch water yesterday? is Yes:

Person9_containers: 99. When [NAME] went to fetch water, which container did [NAME] use? [Estimate container size in Liters]

If 97. Did [NAME] go to fetch water yesterday? is Yes:

Person9_containers_number: 100. Each time [NAME] goes to fetch water, how many containers like this does [NAME] carry and fill?

If 93. Is there another person to add? is Yes:

Person10: 101. Is there another person to add?

◯ Yes
◯ No
◯ Don't know
◯ Declined to state

If 101. Is there another person to add? is Yes:

Person 10

If 101. Is there another person to add? is Yes:

Person10_name: 102. Who else lives in this household? [Record first name only; ask respondent to begin with any other adults in the household, then list all children from oldest to youngest]

If 101. Is there another person to add? is Yes:

Person10_gender: 103. Is [NAME] male or female?

◯ Male
◯ Female
◯ Declined to state

If 101. Is there another person to add? is Yes:

Person10_age: 104. How old is [NAME]?

If 101. Is there another person to add? is Yes:

Person10_water: 105. Did [NAME] go to fetch water yesterday?

- ◯ Yes
- ◯ No
- ◯ Not applicable
- ◯ Declined to state
- ◯ Declined to state

If 105. Did [NAME] go to fetch water yesterday? is Yes:

Person10_trips: 106. How many times did [NAME] go to fetch water yesterday?

If 105. Did [NAME] go to fetch water yesterday? is Yes:

Person10_containers: 107. When [NAME] went to fetch water, which container did [NAME] use? [Estimate container size in Liters]

If 105. Did [NAME] go to fetch water yesterday? is Yes:

Person10_containers_number: 108. Each time [NAME] goes to fetch water, how many containers like this does [NAME] carry and fill?

If 101. Is there another person to add? is Yes:

Person11: 109. Is there another person to add?

- ◯ Yes
- ◯ No
- ◯ Don't know
- ◯ Declined to state

If 109. Is there another person to add? is Yes:

Person 11

If 109. Is there another person to add? is Yes:

Person11_name: 110. Who else lives in this household? [Record first name only; ask respondent to begin with any other adults in the household, then list all children from oldest to youngest]

If 109. Is there another person to add? is Yes:

Person11_gender: 111. Is [NAME] male or female?

◯ Male
◯ Female
◯ Declined to state

If 109. Is there another person to add? is Yes:

Person11_age: 112. How old is [NAME]?

If 109. Is there another person to add? is Yes:

Person11_water: 113. Did [NAME] go to fetch water yesterday?

◯ Yes
◯ No
◯ Not applicable
◯ Declined to state
◯ Declined to state

If 113. Did [NAME] go to fetch water yesterday? is Yes:

Person11_trips: 114. How many times did [NAME] go to fetch water yesterday?

If 113. Did [NAME] go to fetch water yesterday? is Yes:

Person11_containers: 115. When [NAME] went to fetch water, which container did [NAME] use? [Estimate container size in Liters]

If 113. Did [NAME] go to fetch water yesterday? is Yes:

Person11_containers_number: 116. Each time [NAME] goes to fetch water, how many containers like this does [NAME] carry and fill?

If 109. Is there another person to add? is Yes:

Person12: 117. Is there another person to add?

◯ Yes
◯ No
◯ Don't know
◯ Declined to state

If 117. Is there another person to add? is Yes:

Person 12

If 117. Is there another person to add? is Yes:

Person12_name: 118. Who else lives in this household? [Record first name only; ask respondent to begin with any other adults in the household, then list all children from oldest to youngest]

If 117. Is there another person to add? is Yes:

Person12_gender: 119. Is [NAME] male or female?

◯ Male
◯ Female
◯ Declined to state

If 117. Is there another person to add? is Yes:

Person12_age: 120. How old is [NAME]?

If 117. Is there another person to add? is Yes:

Person12_water: 121. Did [NAME] go to fetch water yesterday?

◯ Yes
◯ No
◯ Not applicable
◯ Declined to state
◯ Declined to state

If 121. Did [NAME] go to fetch water yesterday? is Yes:

Person12_trips: 122. How many times did [NAME] go to fetch water yesterday?

If 121. Did [NAME] go to fetch water yesterday? is Yes:

Person12_containers: 123. When [NAME] went to fetch water, which container did [NAME] use? [Estimate container size in Liters]

If 121. Did [NAME] go to fetch water yesterday? is Yes:

Person12_containers_number: 124. Each time [NAME] goes to fetch water, how many containers like this does [NAME] carry and fill?

If 117. Is there another person to add? is Yes:

More_people: 125. Are there any more people to add? How many?

If 14. Has informed consent been obtained? is Yes:

Water Source

Dry_Source_1: 126. What is the main source of drinking-water for members of your household during the dry season?

- ◯ Piped water into dwelling
- ◯ Piped water to yard/plot
- ◯ Public tap/standpipe
- ◯ Borehole
- ◯ Protected dug well
- ◯ Unprotected dug well
- ◯ Protected spring
- ◯ Unprotected spring
- ◯ Rainwater collection
- ◯ Pay another person to fetch/buy filled containers from a vendor
- ◯ Bottled water/sachet water/"pure water"
- ◯ Cart with small tank/drum
- ◯ Tanker truck
- ◯ Surface water (river, dam, lake, pond, stream, canal, irrigation channels)
- ◯ Other (please specify)
- ◯ Not applicable
- ◯ Don't know
- ◯ Declined to state

Water_On_plot_dry: 127. [Direct Observation] Is the household's main dry season water source on-plot?

- ◯ Yes
- ◯ No
- ◯ Not applicable
- ◯ Don't know

Dry_Source_months: 128. For how many months each year do you use this source?

Dry_source_unavailable: 129. Are there ever times during the dry season when water is not available from [SOURCE]?

◯ Yes
◯ No
◯ Declined to state
◯ Not applicable
◯ Don't know

If 129. Are there ever times during the dry season when water is not available from [SOURCE]? is Yes:

Dry_Source_2: 130. When your main source is not available, what other source of drinking-water for members of your household do you use in the dry season?

◯ Piped water into dwelling
◯ Piped water to yard/plot
◯ Public tap/standpipe
◯ Borehole
◯ Protected dug well
◯ Unprotected dug well
◯ Protected spring
◯ Unprotected spring
◯ Rainwater collection
◯ Pay another person to fetch/ buy filled containers from a vendor
◯ Bottled water/sachet water/ "pure water"
◯ Cart with small tank/drum
◯ Tanker-truck
◯ Surface water (river, dam, lake, pond, stream, canal, irrigation channels)
◯ Other (please specify)

◯ Not applicable
◯ Don't know
◯ Declined to state

Wet_Source_1: 131. What is the main source of drinking-water for members of your household during the wet season?

◯ Piped water into dwelling
◯ Piped water to yard/plot
◯ Public tap/standpipe
◯ Borehole
◯ Protected dug well
◯ Unprotected dug well
◯ Protected spring
◯ Unprotected spring
◯ Rainwater collection
◯ Pay another person to fetch/buy filled containers from a vendor
◯ Bottled water/sachet water/"pure water"
◯ Cart with small tank/drum
◯ Tanker truck
◯ Surface water (river, dam, lake, pond, stream, canal, irrigation channels)
◯ Other (please specify)

◯ Not applicable
◯ Don't know
◯ Declined to state

Water_On_plot_wet: 132. [Direct Observation] Is the household's main wet season water source on-plot?

◯ Yes
◯ No
◯ Not applicable
◯ Don't know

Wet_Source_months: 133. For how many months each year do you use this source?

Wet_source_unavailable: 134. Are there ever times during the wet season when water is not available from [SOURCE]?

◯ Yes
◯ No
◯ Not applicable
◯ Don't know
◯ Declined to state

If 134. Are there ever times during the wet season when water is not available from [SOURCE]? is Yes:

Wet_Source_2: 135. When your main source is not available, what other source of drinking-water for members of your household do you use in the wet season?

◯ Piped water into dwelling
◯ Piped water to yard/plot
◯ Public tap/standpipe
◯ Borehole
◯ Protected dug well
◯ Unprotected dug well
◯ Protected spring
◯ Unprotected spring
◯ Rainwater collection
◯ Pay another person to fetch/buy filled containers from a vendor
◯ Bottled water/sachet water/"pure water"
◯ Cart with small tank/drum
◯ Tanker truck
◯ Surface water (river, dam, lake, pond, stream, canal, irrigation channels)
◯ Other (please specify)

____

◯ Not applicable
◯ Don't know
◯ Declined to state

If 14. Has informed consent been obtained? is Yes:

Water reliability

Last_source: 136. What water source did you most recently fetch water from?

- ◯ Piped water into dwelling
- ◯ Piped water to yard/plot
- ◯ Public tap/standpipe
- ◯ Borehole
- ◯ Protected dug well
- ◯ Unprotected dug well
- ◯ Protected spring
- ◯ Unprotected spring
- ◯ Rainwater collection
- ◯ Pay another person to fetch/buy filled containers from a vendor
- ◯ Bottled water/sachet water/"pure water"
- ◯ Cart with small tank/drum
- ◯ Tanker truck
- ◯ Surface water (river, dam, lake, pond, stream, canal, irrigation channels)
- ◯ Other (please specify)

[ ]

- ◯ Not applicable
- ◯ Don't know
- ◯ Declined to state

If 136. What water source did you most recently fetch water from? is one of Piped water into dwelling, Piped water to yard/plot, Public tap/standpipe:

Last_pipe: 137. Is it a municipal piped source or a community piped source?

- ◯ Community piped source
- ◯ Municipal piped source
- ◯ Not applicable
- ◯ Don't know
- ◯ Declined to state

If 136. What water source did you most recently fetch water from? is one of Piped water into dwelling, Piped water to yard/plot, Public tap/standpipe:

Last_pipe_source: 138. What is the source of this piped water? Is it from a mechanized borehole, a protected spring, a surface water source like a river or lake, or somewhere else?

- ◯ Mechanized borehole
- ◯ Protected spring
- ◯ Surface water
- ◯ Not applicable
- ◯ Don't know
- ◯ Declined to state

If 138. What is the source of this piped water? Is it from a mechanized borehole, a protected spring, a surface water source like a river or lake, or somewhere else? is Surface water:

Last_surface: 139. Is this surface water treated in some way before it goes into the pipes?

- ◯ Yes
- ◯ No
- ◯ Don't know
- ◯ Declined to state

Carry_mode: 139.2 How do you normally carry water home from this source?

- ◯ Carry on foot
- ◯ Using a hand pushed cart
- ◯ Using a donkey cart
- ◯ Using a bicycle
- ◯ Using a motorbike
- ◯ Other (please specify)

[ ]

- ◯ Not applicable
- ◯ Don't know
- ◯ Declined to state

Pay_water: 140. Do you have to pay to collect water from this source?

- ◯ Yes
- ◯ No
- ◯ Not applicable
- ◯ Don't know
- ◯ Declined to state

If 140. Do you have to pay to collect water from this source? is Yes:

Pay_water_often: 141. Do you pay as you fetch, or do you pay at certain times every month or year?

- ◯ Every time they fetch
- ◯ Daily
- ◯ Weekly
- ◯ Monthly
- ◯ Yearly
- ◯ When the system breaks
- ◯ No fixed schedule
- ◯ Not applicable
- ◯ Don't know
- ◯ Declined to state

If 141. Do you pay as you fetch, or do you pay at certain times every month or year? is Every time they fetch:

Pay_container: 142. How much do you pay each time to fill the container you showed me earlier? [In CFA]

If 141. Do you pay as you fetch, or do you pay at certain times every month or year? is Daily:

Pay_day: 143. How much do you pay each day? [In CFA]

If 141. Do you pay as you fetch, or do you pay at certain times every month or year? is Weekly:

Pay_week: 144. How much do you pay each week? [in CFA]

If 141. Do you pay as you fetch, or do you pay at certain times every month or year? is Monthly:

Pay_month: 145. How much do you pay each month? [in CFA]

If 141. Do you pay as you fetch, or do you pay at certain times every month or year? is Yearly:

Pay_year: 146. How much do you pay each year? [In CFA]

Water_continuous: 147. Is water available from this source at all times?

◯ Yes
◯ No
◯ Not applicable
◯ Don't know
◯ Declined to state

If 147. Is water available from this source at all times? is No:

Failure_2weeks: 148. Has there been any time in the last two weeks that you could not get any water from [SOURCE] for a full day or more?

◯ Yes
◯ No
◯ Not applicable
◯ Don't know
◯ Declined to state

If 148. Has there been any time in the last two weeks that you could not get any water from [SOURCE] for a full day or more? is Yes:

Reason_nowater: 149. Why were you unable to get water from this source?

◯ No water available at source
◯ Source locked
◯ Physically unable to fetch
◯ Unable to pay
◯ Other (please specify)

____

◯ Don't know
◯ Declined to state

If 148. Has there been any time in the last two weeks that you could not get any water from [SOURCE] for a full day or more? is Yes:

Failure_days: 150. For how many days in the last two weeks was water not available?

____

If 148. Has there been any time in the last two weeks that you could not get any water from [SOURCE] for a full day or more? is Yes:

Predict_days: 151. Are you able to predict which days water will be available from this source?

◯ Yes
◯ No
◯ Not applicable
◯ Don't know
◯ Declined to state

If 147. Is water available from this source at all times? is No:

Water_hours: 152. Is water available from this source at all hours of the day?

◯ Yes
◯ No
◯ Not applicable
◯ Don't know
◯ Declined to state

If 152. Is water available from this source at all hours of the day? is No:

Predict_hours: 153. Are you able to predict which hours water will be available from this source?

◯ Yes
◯ No
◯ Not applicable
◯ Don't know
◯ Declined to state

Seaonality: 154. Are there months during the year that water is not available from this source?

◯ Yes
◯ No
◯ Not applicable
◯ Don't know
◯ Declined to state

If 154. Are there months during the year that water is not available from this source? is Yes:

Months: 155. Which month(s) is water not available from this source? [Mark all that apply]

- ☐ January
- ☐ February
- ☐ March
- ☐ April
- ☐ May
- ☐ June
- ☐ July
- ☐ August
- ☐ September
- ☐ October
- ☐ November
- ☐ December
- ☐ Not applicable
- ☐ Don't know
- ☐ Declined to state

If 14. Has informed consent been obtained? is Yes:

Water Functionality

Water_failure: 156. Has there been any time in the last year that you could not get any water from [source] for a full day or more (including today)?

- ◯ Yes
- ◯ No
- ◯ Declined to state
- ◯ Not applicable
- ◯ Don't know

If 156. Has there been any time in the last year that you could not get any water from [source] for a full day or more (including today)? is Yes:

Water_downtime: 157. For how long was water not available from your main source the last time it broke down? [If system is still broken, record time since the system broke.]

If 156. Has there been any time in the last year that you could not get any water from [source] for a full day or more (including today)? is Yes:

Breakdown_number: 158. How many times has this water point broken down in the past year?

If 14. Has informed consent been obtained? is Yes:

Multiple Uses of Water

Water_farm: 159. Do you use water from your main water source for a farm or garden?

◯ Farm
◯ Garden
◯ Both
◯ None
◯ Not applicable
◯ Don't know
◯ Declined to state

Water_business: 160. Do you use your main water source for a business? [If yes, ask what type of business: mark all that apply]

☐ No
☐ Restaurant
☐ Prepared food or drinks
☐ Washing cars
☐ Washing clothes for money
☐ Construction
☐ Fetching water for others
☐ Other (please specify)

☐ Don't know
☐ Declined to state

If 14. Has informed consent been obtained? is Yes:

Water Accessibility

Source_visit: 161. Can you take me to the water source that you most recently fetched water from?

◯ Yes
◯ No
◯ Sends other person to show water source

If 161. Can you take me to the water source that you most recently fetched water from? is one of Yes, Sends other person to show water source:

Source_start_hour: 162. Record time you start water walk: Hour

Hint: Based on 24 hour clock

If 161. Can you take me to the water source that you most recently fetched water from? is one of Yes, Sends other person to show water source:

Source_start_min: 163. Record time you start water walk: Minute

If 161. Can you take me to the water source that you most recently fetched water from? is one of Yes, Sends other person to show water source:

Source_arr_hour: 164. Record time arrival to water point: Hour

Hint: Based on 24 hour clock

If 161. Can you take me to the water source that you most recently fetched water from? is one of Yes, Sends other person to show water source:

Source_arr_min: 165. Record time of arrival to water point: Minute

If 161. Can you take me to the water source that you most recently fetched water from? is one of Yes, Sends other person to show water source:

water_geo_code: 166. Record GPS coordinates of water point

Hint: Wait for GPS within 10 m accuracy

If 161. Can you take me to the water source that you most recently fetched water from? is one of Yes, Sends other person to show water source:

Water_ID: 166.2 Water source ID (if present)

If 161. Can you take me to the water source that you most recently fetched water from? is one of Yes, Sends other person to show water source:

Source_end_hour: 167. [After taking GPS coordinates, return to the house] Record time of return: hour

Hint: Based on 24 hour clock

If 161. Can you take me to the water source that you most recently fetched water from? is one of Yes, Sends other person to show water source:

Source_end_min: 168. Record time of return: minute

Wait_time: 169.1 How long do you usually have to queue to fetch water from this source in the dry season?

____

Queue_wet: 169.2 How long do you usually have to queue to fetch water from this source in the wet season?

____

If 14. Has informed consent been obtained? is Yes:

Household water

Treat: 170. Do you treat your water to make it safer for drinking?

◯ Yes
◯ No
◯ Don't know
◯ Declined to state

If 170. Do you treat your water to make it safer for drinking? is Yes:

Treat_type: 171. What do you usually do to the water to make it safer to drink? Anything else? [Do not read choices, mark all items mentioned]

☐ Boil
☐ Add bleach/chlorine
☐ Strain it through a cloth
☐ Use a water filter (ceramic or sand or composite, etc)
☐ Solar disinfection
☐ Let it stand and settle
☐ Other (please specify)

____

☐ Don't know
☐ Declined to state

If 170. Do you treat your water to make it safer for drinking? is Yes:

Treat_frequency: 172. In the last two weeks, have you treated your water:

◯ Everyday
◯ Most of the days
◯ Half of the days
◯ Less than half of the days
◯ Not at all
◯ Don't know
◯ Declined to state

☐ Don't Know

Sample_ID1: 173. Water sample ID

Sample_ID: 174. Water sample ID (confirm)

Storage_char: 175. Can you serve me some water the way you normally take it? [Direct Observation] Does/is the drinking-water storage container: [Mark all that apply]

☐ Have a lid that is completely covering it
☐ Have a narrow opening
☐ Have a tap or spigot
☐ Beyond reach of animals (1 meter or more from the ground)
☐ Clean (free of dirt- debris- garbage- faecal matter- etc.)
☐ Does not have container
☐ Other (please specify)

☐ Declined to show
☐ None of the above

Method_serve: 176. [Photo] Take a picture of the respondent taking water from the drinking-water storage container the way they normally take it

Utensil: 177. [Direct Observation] What was used to take water from the storage container?

◯ Nothing (water poured or dispensed through a spigot or spout)
◯ Dipper or ladle
◯ Bucket
◯ Hand
◯ Cup or bowl or jar or can
◯ Other (please specify)

____

◯ Not applicable

178. What is the source of this [the water that is sampled] water? [Mark all that apply]

☐ Piped water into dwelling
☐ Piped water to yard/plot
☐ Public tap/standpipe
☐ Borehole
☐ Protected dug well
☐ Unprotected dug well
☐ Protected spring
☐ Unprotected spring
☐ Rainwater collection
☐ Pay another person to fetch/buy filled containers from a vendor
☐ Bottled water/sachet water/"pure water"
☐ Cart with small tank/drum
☐ Tanker truck
☐ Surface water (river, dam, lake, pond, stream, canal, irrigation channels)
☐ Other (please specify)

____

☐ Not applicable
☐ Don't know
☐ Declined to state

Floor: 179. [Direct Observation] What is the main material of the floors inside all the rooms of the house?

- ◯ Earth/sand
- ◯ Dung
- ◯ Wood planks
- ◯ Palm/bamboo
- ◯ Parquet or polished wood
- ◯ Vinyl or asphalt strips
- ◯ Ceramic tiles
- ◯ Cement
- ◯ Carpet
- ◯ Other (please specify)

____

- ◯ Don't know

Walls: 180. [Direct Observation] What is the main material of the dwelling walls?

- ◯ No walls
- ◯ Dirt/earth
- ◯ Cement
- ◯ Dung
- ◯ Cane/Palm/Tree trunks
- ◯ Bamboo with mud
- ◯ Stone with mud
- ◯ Uncovered adobe
- ◯ Plywood
- ◯ Cardboard
- ◯ Reused wood
- ◯ Stone with lime/cement
- ◯ Bricks
- ◯ Cement blocks
- ◯ Covered adobe
- ◯ Wood planks/shingles
- ◯ Other (please specify)

____

- ◯ Don't know

Roof: 181. [Direct Observation] What is the main material of the dwelling roof?

◯ No roof
◯ Thatch/Straw/Palm leaf
◯ Metal
◯ Wood planks
◯ Sod/grass and earth
◯ Rustic mat/woven plant material
◯ Palm/bamboo
◯ Cardboard
◯ Finished wood boards
◯ Calamine/cement fibre
◯ Ceramic tiles
◯ Cement slab
◯ Roofing shingles
◯ Plastic
◯ Other (please specify)

◯ Don't know

If 14. Has informed consent been obtained? is Yes:

Sanitation facility

Defecate1: 182. [Do not read answers out loud] Some people prefer to defecate in the bush or the open, some prefer to defecate in a latrine, and some prefer other places. What are the places that adult men and women in this household defecate? [Mark all that apply] Probe to ask "Is there any other place?" until they finish

☐ [Latrine]
☐ [Bush/field/no sanitation facility]
☐ [In water body (river or lake)]
☐ Other (please specify)

☐ Don't know
☐ Declined to state

Defecate2: 183. [Do not read answers out loud] Some people prefer to defecate in the bush or the open, some prefer to defecate in a latrine, and some prefer other places. Where are the places that boys and girls over the age of 3 in this household go to defecate [Mark all that apply] Probe to ask "Is there any other place?" until they finish

☐ [Latrine]
☐ [Bush/field/no sanitation facility]
☐ [In water body (river or lake)]
☐ Other (please specify)

☐ Not applicable
☐ Don't know
☐ Declined to state

Latrine_defecate: 184. [Direct Observation] According to the answers of the two previous questions, does anyone in this household defecate in a latrine

◯ Yes
◯ No
◯ Not applicable
◯ Don't know
◯ Declined to state

If 184. [Direct Observation] According to the answers of the two previous questions, does anyone in this household defecate in a latrine is Yes:

San_visit: 185. Can you show me the toilet facility that you use?

◯ Yes
◯ No
◯ Sends another person to show sanitation facility

If 185. Can you show me the toilet facility that you use? is one of Yes, Sends another person to show sanitation facility:

San_start_hour: 186. Record time you leave for toilet facility: hour

Hint: Using a 24 hour clock

If 185. Can you show me the toilet facility that you use? is one of Yes, Sends another person to show sanitation facility:

San_start_min: 187. Record time you leave for toilet facility: minute

If 185. Can you show me the toilet facility that you use? is one of Yes, Sends another person to show sanitation facility:

San_arrive_hr: 187.1 Record time you arrive at the toilet facility: hour

If 185. Can you show me the toilet facility that you use? is one of Yes, Sends another person to show sanitation facility:

San_arrive_min: 187.2 Record time you arrive at the toilet facility: minute

If 185. Can you show me the toilet facility that you use? is one of Yes, Sends another person to show sanitation facility:

Sanitation_ID: 188. Sanitation facility ID

If 185. Can you show me the toilet facility that you use? is one of Yes, Sends another person to show sanitation facility:

San_geo_code: 189. [Direct Observation] GPS coordinates of primary sanitation facility

Hint: Wait until GPS coordinates within 10m accuracy

If 185. Can you show me the toilet facility that you use? is one of Yes, Sends another person to show sanitation facility:

San_location: 190. [Direct Observation] Is the sanitation-facility on-plot?

◯ Yes, in own dwelling
◯ Yes, in own yard/plot
◯ No, facility is off-plot
◯ Not observed

If 185. Can you show me the toilet facility that you use? is one of Yes, Sends another person to show sanitation facility:

San_type_on_plot: 191. [Direct Observation] What type of toilet facility is it? [If “flush” or “pour-flush” and you cannot tell where the waste goes, probe] Where does it flush to?*

- ◯ Flush/pour flush to piped sewer system
- ◯ Flush/pour flush to piped septic tank
- ◯ Flush/pour flush to pit latrine
- ◯ Flush/pour flush to elsewhere
- ◯ Flush/pour flush to unknown place/not sure/don't know
- ◯ Ventilated improved pit latrine (VIP)
- ◯ Pit latrine with slab
- ◯ Pit latrine without slab/open pit
- ◯ Composting toilet
- ◯ Bucket
- ◯ Hanging toilet/hanging latrine
- ◯ Other (please specify)

____

- ◯ Don't know

If 190. [Direct Observation] Is the sanitation-facility on-plot? is one of No, facility is off-plot, Not observed:

San_type_off_plot: 192. [Direct Observation] What type of toilet facility is it? [If “flush” or “pour-flush” and you cannot tell where the waste goes, probe] Where does it flush to?

- ◯ Flush/pour flush to piped sewer system
- ◯ Flush/pour flush to piped septic tank
- ◯ Flush/pour flush to pit latrine
- ◯ Flush/pour flush to elsewhere
- ◯ Flush/pour flush to unknown place/not sure/don't know
- ◯ Ventilated improved pit latrine (VIP)
- ◯ Pit latrine with slab
- ◯ Pit latrine without slab/open pit
- ◯ Composting toilet
- ◯ Bucket
- ◯ Hanging toilet/hanging latrine
- ◯ Other (please specify)

____

- ◯ Don't know

If 185. Can you show me the toilet facility that you use? is one of Yes, Sends another person to show sanitation facility:

San_use: 193. [Direct Observation] Does the facility shows signs of recent use?

◯ Yes
◯ No
◯ Don't know

If 185. Can you show me the toilet facility that you use? is one of Yes, Sends another person to show sanitation facility:

San_accessible: 194. [Direct Observation] Is this facility accessible to disabled people?

◯ Yes
◯ No
◯ Don't know

If 185. Can you show me the toilet facility that you use? is one of Yes, Sends another person to show sanitation facility:

San_image: 195. [Photo] Take a photo of the sanitation facility

If 185. Can you show me the toilet facility that you use? is one of Yes, Sends another person to show sanitation facility:

San_functional: 196. [Direct Observation] Is the sanitation facility in working order?

◯ In working order
◯ Not in working order
◯ Don't know

If 196. [Direct Observation] Is the sanitation facility in working order? is Not in working order:

San_nonfunctional: 197. [Direct Observation] Why is the sanitation facility not functioning as intended?

◯ Facilities unreliable
◯ Facilities unhygienic
◯ Facilities poorly constructed
◯ Pit full
◯ Facilities flooded
◯ No water
◯ Locked
◯ Other (please specify)

◯ Don't know

If 185. Can you show me the toilet facility that you use? is one of Yes, Sends another person to show sanitation facility:

San_Condition_1: 198. [Direct Observation] Is the inside of the sanitation facility soiled with feces?

◯ Yes
◯ No
◯ Don't know

If 185. Can you show me the toilet facility that you use? is one of Yes, Sends another person to show sanitation facility:

San_Condition_2: 199. [Direct Observation] Is there evidence of feces on the ground within 10 meters of the sanitation facility?

◯ Yes
◯ No
◯ Don't know

If 185. Can you show me the toilet facility that you use? is one of Yes, Sends another person to show sanitation facility:

San_Condition_3: 200. [Direct Observation] Is there an unpleasant or offensive smell within the sanitation facility which could discourage use of the facility.

◯ Yes
◯ No
◯ Don't know

If 191. [Direct Observation] What type of toilet facility is it? [If "flush" or "pour-flush" and you cannot tell where the waste goes, probe] Where does it flush to? is one of Flush/pour flush to piped sewer system, Flush/pour flush to piped septic tank, Flush/pour flush to pit latrine, Flush/pour flush to elsewhere, Flush/pour flush to unknown place/not sure/don't know, Ventilated improved pit latrine (VIP), Pit latrine with slab, Pit latrine without slab/open pit, Composting toilet:*

San_Condition_4: 201. [Direct Observation] Is there evidence of cracking or damage to the toilet pedestal or squat-slab?

◯ Yes
◯ No
◯ Don't know

If 191. [Direct Observation] What type of toilet facility is it? [If "flush" or "pour-flush" and you cannot tell where the waste goes, probe] Where does it flush to? is one of Flush/pour flush to piped sewer system, Flush/pour flush to piped septic tank, Flush/pour flush to pit latrine, Flush/pour flush to elsewhere, Flush/pour flush to unknown place/not sure/don't know, Ventilated improved pit latrine (VIP), Pit latrine with slab, Pit latrine without slab/open pit, Composting toilet:*

San_Condition_5: 202. [Direct Observation] Is there any damage to the pipes or plumbing?

◯ Yes
◯ No
◯ Don't know

If 191. [Direct Observation] What type of toilet facility is it? [If “flush” or “pour-flush” and you cannot tell where the waste goes, probe] Where does it flush to? is one of Flush/pour flush to pit latrine, Ventilated improved pit latrine (VIP), Pit latrine with slab, Pit latrine without slab/open pit, Composting toilet:*

San_Condition_6: 203. [Direct Observation] Is the pit uncovered?

◯ Yes
◯ No
◯ Don't know

If 191. [Direct Observation] What type of toilet facility is it? [If “flush” or “pour-flush” and you cannot tell where the waste goes, probe] Where does it flush to? is one of Ventilated improved pit latrine (VIP), Pit latrine with slab, Pit latrine without slab/open pit, Flush/pour flush to pit latrine, Composting toilet:*

San_Condition_7: 204. [Direct Observation] Is the cover slab incompletely sealed?

◯ Yes
◯ No
◯ Not applicable
◯ Don't know

If 191. [Direct Observation] What type of toilet facility is it? [If “flush” or “pour-flush” and you cannot tell where the waste goes, probe] Where does it flush to? is one of Flush/pour flush to piped sewer system, Flush/pour flush to piped septic tank, Flush/pour flush to pit latrine, Ventilated improved pit latrine (VIP), Pit latrine with slab, Pit latrine without slab/open pit, Composting toilet:*

San_Condition_8: 205. [Direct Observation] Is there evidence that the pit or septic tank is full, overflowing or allowing wastes to leak onto the ground?

◯ Yes
◯ No
◯ Don't know

If 191. [Direct Observation] What type of toilet facility is it? [If “flush” or “pour-flush” and you cannot tell where the waste goes, probe] Where does it flush to? is one of Flush/pour flush to elsewhere, Flush/pour flush to unknown place/not sure/don't know:*

San_Condition_9: 206. [Direct Observation] Are excreta discharged directly to the ground or to an open sewer or gutter?

◯ Directly to the ground
◯ To an open sewer or gutter
◯ Other (please specify)

____

◯ Don't know

If 185. Can you show me the toilet facility that you use? is Yes:

San_construct: 207. When was this toilet facility constructed?

____

If 185. Can you show me the toilet facility that you use? is Yes:

Latrine_overflow: 208. Has this toilet facility ever completely filled with excreta so that it was unusable or overflowing?

◯ Yes
◯ No
◯ Declined to state
◯ Not applicable
◯ Don't know

If 185. Can you show me the toilet facility that you use? is No:

San_type_ask: 209. What type of toilet facility do you use? [If "flush" or "pour-flush" probe] Where does it flush to?

◯ Flush/pour flush to piped sewer system
◯ Flush/pour flush to piped septic tank
◯ Flush/pour flush to pit latrine
◯ Flush/pour flush to elsewhere
◯ Flush/pour flush to unknown place/not sure/don't know
◯ Ventilated improved pit latrine (VIP)
◯ Pit latrine with slab
◯ Pit latrine without slab/open pit
◯ Composting toilet
◯ Bucket
◯ Hanging toilet/hanging latrine
◯ No facilities or bush or field
◯ Other (please specify)

____

◯ Declined to state

If 184. [Direct Observation] According to the answers of the two previous questions, does anyone in this household defecate in a latrine is Yes:

San_shared: 210. Is this facility shared with other families who are not relatives?

◯ Yes
◯ No
◯ Declined to state

☐ Don't Know
☐ Not Applicable

If 184. [Direct Observation] According to the answers of the two previous questions, does anyone in this household defecate in a latrine is Yes:

San_house_num: 211. How many households (including your own) use this facility?

If 184. [Direct Observation] According to the answers of the two previous questions, does anyone in this household defecate in a latrine is Yes:

San_functional: 212. Is the sanitation facility for your household functional?

◯ Yes
◯ No
◯ Don't know
◯ Declined to state

If 184. [Direct Observation] According to the answers of the two previous questions, does anyone in this household defecate in a latrine is Yes:

San_use_ask: 213. Is your household using this sanitation facility?

◯ Yes
◯ No
◯ Don't know
◯ Declined to state

If 185. Can you show me the toilet facility that you use? is one of Yes, Sends another person to show sanitation facility:

San_end_hour: 214. Record time you return from sanitation walk: hour

Hint: Using a 24 hour clock

If 185. Can you show me the toilet facility that you use? is one of Yes, Sends another person to show sanitation facility:

San_end_min: 215. Record time you return from sanitation walk: minute

If 14. Has informed consent been obtained? is Yes:

Sanitation Reliability

San_accessibility: 216. Are you able to use this facility at all times?

◯ Yes
◯ No
◯ Not applicable
◯ Don't know
◯ Declined to state

If 216. Are you able to use this facility at all times? is No:

San_hr: 217. During what hours are you unable to use the sanitation facility?

Hint: Using a 24 hour clock

If 216. Are you able to use this facility at all times? is No:

San_whyno: 218. Why were you unable to use your sanitation facility?

- ◯ Pit became full
- ◯ Pit collapsed
- ◯ Structure collapsed
- ◯ Pit became flooded
- ◯ Pipe became blocked
- ◯ Facility too dirty
- ◯ Smell too unpleasant
- ◯ Fear of animals or snakes
- ◯ Facility too hot to use
- ◯ No water
- ◯ Concern for safety
- ◯ Facility locked
- ◯ Other (please specify)
- ◯ Don't know
- ◯ Declined to state

San_breakdown: 219. In the past year, has the sanitation facility been not available or out of service for more than one day?

- ◯ Yes
- ◯ No
- ◯ Declined to state
- ◯ Not applicable
- ◯ Don't know

If 219. In the past year, has the sanitation facility been not available or out of service for more than one day? is Yes:

San_breakdown_time: 220. How long was the sanitation facility out of service the last time it broke down? [If system is still broken, record time since the system broke.]

If 219. In the past year, has the sanitation facility been not available or out of service for more than one day? is Yes:

San_unusable: 221. Why were you unable to use your sanitation facility?

- ◯ Pit became full
- ◯ Pit collapsed
- ◯ Structure collapsed
- ◯ Pit became flooded
- ◯ Pipe became blocked
- ◯ Facility too dirty
- ◯ Smell too unpleasant
- ◯ Fear of animals or snakes
- ◯ Facility too hot to use
- ◯ No water
- ◯ Concern for safety
- ◯ Facility locked
- ◯ Other (please specify)

____

- ◯ Don't know
- ◯ Declined to state

If 14. Has informed consent been obtained? is Yes:

Sanitation excreta disposal

Child_under3: 222. Does any child younger than 3 years old live in this household?

- ◯ Yes
- ◯ No
- ◯ Declined to state
- ◯ Don't know

If 222. Does any child younger than 3 years old live in this household? is Yes:

Child_feces: 223. [Do not read answer choices] The last time the youngest child (less than 3 years) passed feces, what was done to dispose of the feces?

◯ [Child used toilet/latrine]
◯ [Put/rinsed into toilet or latrine]
◯ [Put/rinsed into drain or ditch]
◯ [Thrown into garbage bin or pile]
◯ [Buried]
◯ [Threw feces away in the open/threw in bush]
◯ [Left in the open]
◯ Other (please specify)

◯ Don't know
◯ Declined to state

Open_defecation: 224. Have you seen a person openly defecate in this community in the past two weeks?

◯ Yes
◯ No
◯ Don't know
◯ Declined to state

If 14. Has informed consent been obtained? is Yes:

Hygiene

Show_hyg: 225. Can you show me how you wash your hands? [Direct Observation] How does the respondent wash their hands? [Mark all that apply]

☐ [Use of water]
☐ [Use of soap]
☐ [Use of ash or other cleanser]
☐ [Rubbing motion]
☐ Not shown

Hgy_supplies: 226. [Direct Observation] Are soap (or another cleanser) and water present in the household?

◯ Present (observed)
◯ Not present (observed)

If 226. [Direct Observation] Are soap (or another cleanser) and water present in the household? is Present (observed):

Soaptype: 227. [Direct Observation] What type of detergent or cleanser is used? [Mark all that apply]

☐ [Soap]
☐ [Ash]
☐ [Mud or sand (specifically for hand hygiene)]
☐ None
☐ Other (please specify)

Hyg_location: 228. [Direct Observation] Is there a fixed location for handwashing?

◯ Yes
◯ No
◯ Not observed

If 228. [Direct Observation] Is there a fixed location for handwashing? is Yes:

Hyg_geo_code: 229. [Direct Observation] GPS coordinates of hygiene location

When_wash1: 230. [Do not read the options] When do you wash your hands? [Mark all that apply]

☐ [After defecation]
☐ [After cleaning or changing a baby]
☐ [Before food preparation]
☐ [Before eating]
☐ [Before feeding a child]
☐ Other (please specify)

☐ Don't know
☐ Declined to state

When_wash2: 231. [Do not read the options] Are there any other times that you wash your hands? [Mark all that apply]

☐ [After defecation]
☐ [After cleaning or changing a baby]
☐ [Before food preparation]
☐ [Before eating]
☐ [Before feeding a child]
☐ Other (please specify)

☐ Don't know
☐ Declined to state

If 14. Has informed consent been obtained? is Yes:

Household Characteristics II

Occupation: 232. What is the primary occupation of the highest-earning member of your household (including yourself)?

◯ No occupation
◯ Farming
◯ Raising livestock to sell
◯ Labor or construction
◯ Selling agricultural goods
◯ Selling other goods
◯ Teaching
◯ Office worker
◯ Secretary
◯ Government employee/civil servant
◯ Driver
◯ Craftsman (carpenter- metal worker- electrician- etc.)
◯ Banking- finance
◯ Owns a food stall or restaurant
◯ Selling food
◯ Owns a business that is not a farm or restaurant
◯ Pastor or other religious position
◯ Other (please specify)

◯ Don't know
◯ Declined to state

Bank: 233. Does any member of this household have a bank account?

◯ Yes
◯ No
◯ Don't know
◯ Declined to state

Home: 234. Do you or someone living in this household own this dwelling? If "no", then ask: do you rent this dwelling from someone not living in this household?

◯ Own
◯ Rent
◯ Neither own nor rent
◯ Don't know
◯ Declined to state
◯ Not applicable

Livestock: 235. Does this household own any livestock, herds, other farm animals, or poultry?

◯ Yes
◯ No
◯ Don't know
◯ Declined to state

If 235. Does this household own any livestock, herds, other farm animals, or poultry? is Yes:
Livestock_type: 236. Which animals do you own? [Mark all that apply]

☐ Cows
☐ Goats
☐ Sheep
☐ Chickens/Guinea Fowl/poultry
☐ Other (please specify)

☐ Don't know
☐ Declined to state

Land: 237. Does any member of this household own any land that can be used for agriculture?

◯ Yes
◯ No
◯ Don't know
◯ Declined to state

If 237. Does any member of this household own any land that can be used for agriculture? is Yes:

Land_area: 238. How much land does this household own?

Electricity: 239. Does this house have electricity?

- ◯ Yes
- ◯ No
- ◯ Don't know
- ◯ Declined to state

Stove: 240. What type of fuel does your household mainly use for cooking?

- ◯ Charcoal
- ◯ Wood
- ◯ Straw/Shrubs/Grass
- ◯ Electricity (electric stove)
- ◯ Liquefied Petroleum Gas (LPG)
- ◯ Natural gas
- ◯ Biogas
- ◯ Kerosene
- ◯ Coal / Lignite
- ◯ Animal dung
- ◯ Agricultural crop residue
- ◯ No food cooked in household
- ◯ Don't know
- ◯ Declined to state

Bicycle: 241. Does any member of your household own: A working bicycle?

- ◯ Yes
- ◯ No
- ◯ Don't know
- ◯ Declined to state

Motorbike: 242. Does any member of your household own: A working motobike?

- ◯ Yes
- ◯ No
- ◯ Don't know
- ◯ Declined to state

Car: 243. Does any member of your household own: A working car?

◯ Yes
◯ No
◯ Don't know
◯ Declined to state

Mobile_telephone: 244. Does any member of your household own: A working mobile telephone?

◯ Yes
◯ No
◯ Don't know
◯ Declined to state

Radio: 245. Does any member of your household own: A working radio?

◯ Yes
◯ No
◯ Don't know
◯ Declined to state

Television: 246. Does any member of your household own: A working television?

◯ Yes
◯ No
◯ Don't know
◯ Declined to state

Refrigerator: 247. Does any member of your household own: A working refrigerator?

◯ Yes
◯ No
◯ Don't know
◯ Declined to state

Excreta: 248. [Direct Observation] Are excreta present in the house or yard?

◯ Yes
◯ No
◯ Don't know

Untitled Section

Notes: 249. Thank the respondent for their time [Record your notes here]

End_hour: 250. End time: hour

Hint: Enter hour based on 24 clock

End_min: 251. End time: minute
