# Supplementary material for: Adapting a safe water storage container to improve household stored water quality in rural Burkina Faso: a cluster randomized trial
Source: J Water Sanit Hyg Dev. Author manuscript; Available in PMC 2025 Sep 23. (PMC12453111; doi:10.2166/washdev.2021.065)
Supplement: SI file 3 [file NIHMS2111177-supplement-SI_file_3.docx]

WaterAid – CQI Project : Key informant interview (Storage Container manufacturers)

**Introduction**: WaterAid Burkina Faso and the Water Institute at the University of North Carolina are working to improve the safety of water that is collected and stored in containers by households. As part of the project, we have conducted a study to identify container characteristics that improve the safety of water. Based on our results, we have identified and designed a few potential storage containers to be used by households. We would like to gather some information about the manufacturing of water storage containers. The interview will take between 10-20 minutes and we will not ask any personal information. We will record your name and contact information so that we can contact you if we have additional questions, but we will not distribute your contact information. You can choose not to answer any of the questions or end the interview at any time. There are no anticipated risks in participating in this interview and you will not receive any compensation for your participation. We thank you for your cooperation.

**Name of manufacturer:**

**Contact information (phone):**

**Location of interview:**

Design 1 – Ghana design

1. Where could you order the needed supplies to manufacture this design?

(Name and location of supplier(s))

1. How long do you think it would take to receive the supplies after ordering them?
2. How many days would it take you to manufacture 20 containers? (minimum-maximum)
3. How many days would it take you to manufacture 80 containers? (minimum-maximum)
4. How much would the unit price be for this container?
5. How much would it cost for just the container? (CFA)
6. How much would it cost for just the stand? (CFA)
7. How much would you charge to manufacture 50 containers? (In CFA)
8. How much would you charge to manufacture 80 containers? (In CFA)
9. How much would your lowest rate be for a bulk order? (In CFA)
10. How long do you think this container would last?
11. Do you see any problems in manufacturing this design?
12. Are you interested in manufacturing this kind of container beyond just a special order?

**Design 2 – Jerry can**

1. Where could you order the needed supplies to manufacture this design?

(Name and location of supplier(s))

1. How long do you think it would take to receive the supplies after ordering them?
2. How many days would it take you to manufacture 20 containers? (minimum-maximum)
3. How many days would it take you to manufacture 80 containers? (minimum-maximum)
4. How much would the unit price be for this container? (CFA)
5. How much would it cost for just the tap? (CFA)
6. How much would it cost for just the container? (CFA)
7. How much would it cost for just the stand? (CFA)
8. How much would you charge to manufacture 50 containers? (In CFA)
9. How much would you charge to manufacture 80 containers? (In CFA)
10. How much would your lowest rate be for a bulk order? (In CFA)
11. How long do you think this container would last?
12. Do you see any problems in manufacturing this design?
13. Are you interested in manufacturing this kind of container beyond just a special order?

**Additional comments from the interview:**

**CLOSING**

On behalf of WaterAid Burkina Faso, the Water Institute at UNC and our partners, we thank you for providing answers. If you have additional questions, please feel free to contact Hermann Kambou at the WaterAid Office in Ouagadougou. (Phone - )
