# Supplementary material for: Adapting a safe water storage container to improve household stored water quality in rural Burkina Faso: a cluster randomized trial
Source: J Water Sanit Hyg Dev. Author manuscript; Available in PMC 2025 Sep 23. (PMC12453111; doi:10.2166/washdev.2021.065)
Supplement: SI file 4 [file NIHMS2111177-supplement-SI_file_4.docx]

# Intervention hardware – final design

Intervention hardware consisted of a 40 liter capacity plastic storage container and metal stand (Figure 1). Containers featured a removable lid that could be latched and locked (Figure 2), with a guard integrated into the lid to prevent insertion of hands, cups, ladles, or other objects for scooping water that could introduce contamination to the container (Figure 3). Instead, water was dispensed by a tap at the bottom of the container.

Metal stands elevated the container one meter off the ground. Containers rested on top held in place by a metal ring around the circumference of the container. Containers could be removed as needed for cleaning or other purposes.

Containers and stands were produced at a total cost of 18,000 West African Franks (USD 31) (Table 1).


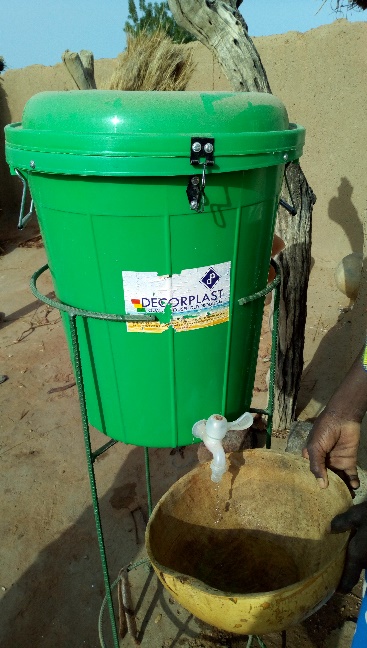


Figure 1. Safe water storage design featuring plastic container with lid and tap mounted on metal stand.


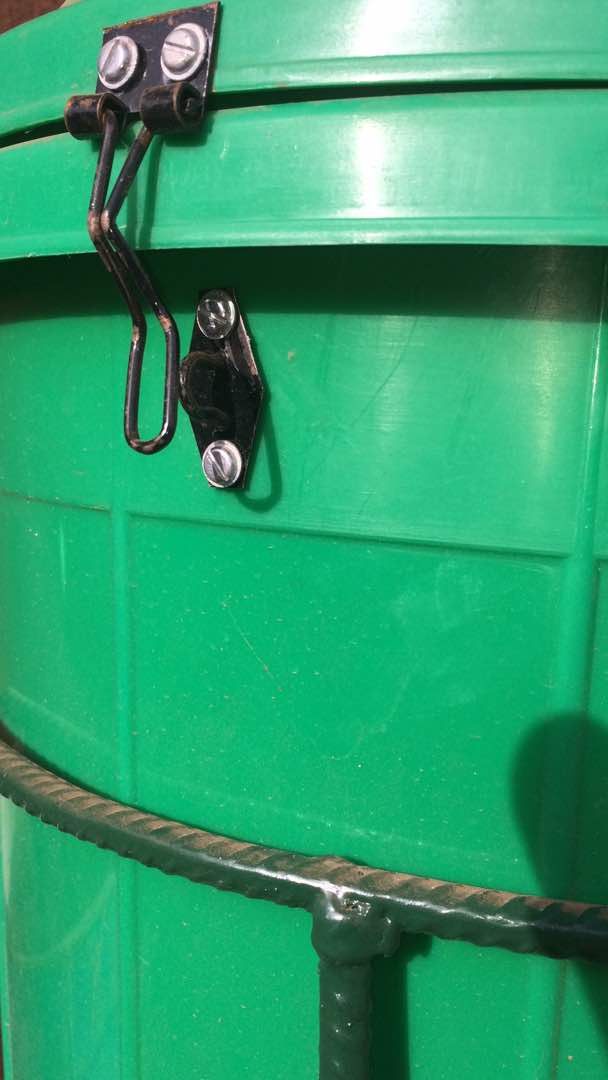


Figure 2. Latch closure on container lid.


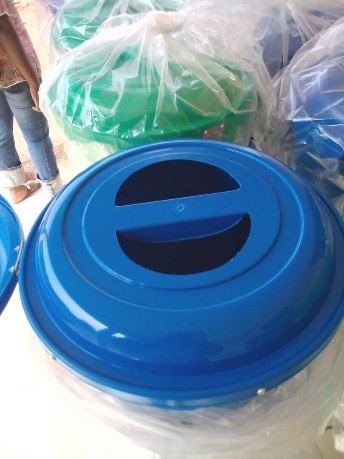


Figure 3. Plastic guard in container lid to prevent insertation of hands or other objects.

Table 1. Cost of intervention hardware components

| **Item** | **Quantity** | **Unit price (CFA)** | **Total price (CFA)** |
| --- | --- | --- | --- |
| Metal stand | 1 | 11 000 | 11 000 |
| 40l bucket with tap and lid | 1 | 7 000 | 7 000 |
| **Total cost** | | | **18 000** |

# Intervention software – final design

Implementers used the following protocols for messaging when delivering safe water storage containers to households.

**Instructions to household users of plastic water storage containers**

1. The household does not have to accept the container, but it is theirs to use for drinking water storage if they want it.
2. The container is being given to those households that were randomly selected for survey questions about water and sanitation earlier.
3. It is designed to keep the water safe so that no contamination occurs, in order to help prevent illness.
4. It only works if water is always fetched using the tap— users should never serve water by dipping in from the top.
5. The container must be kept covered at all times, except when it is being filled.
6. The container should be kept filled with water from a safe source, such as a borehole or a piped supply.
7. All members of the household should drink exclusively from the safe storage container when at home; they should refrain from drinking from other containers without lids and taps, as these are more likely to become contaminated.
8. Children who are old enough to drink water should also drink exclusively from this container when at home. If they are not able to fetch from this container themselves, an adult should serve them water from this container when they want to drink.
9. The UNC enumerators will return to ask more survey questions about water, sanitation, and hygiene in the future. The family should answer as honestly as possible so that we can learn if the safe water storage containers are working as intended.
10. Once the study is finished, if the results indicate that the containers are working as they should, it may help WaterAid learn how to better ensure that communities have safe drinking water in the future.
11. Cleaning should be done no more than once per month. You should be sure not to insert bare hands into the container when cleaning— you can fill part way with water, add two capfuls of chlorine if available, and scrub with a clean brush, if they have one. Otherwise, a clean piece of cloth can be used for cleaning.
12. Thank the household for their cooperation.

# Implementation protocols

Implementers used the following protocols when delivering safe water storage containers to communities.

## Distribution protocol for water storage vessels

This protocol is intended facilitate the distribution of water storage vessels to CQI intervention communities. The distribution will be in two phases in order to afford consistent monitoring and evaluation.

1. **Pre-distribution phase.**
2. Identify the CQI intervention community.
3. Adequate information about the community should have been gathered through a community survey, sanitation survey, water point survey, and surveys of randomly selected households.
4. Extract the GPS coordinates of intervention households (randomly selected) from household survey data set.
5. Plan the day/date and time of distribution of the vessels. Take into consideration, farming activities, market days and important local festivities. Plan to distribute in the period you are most likely to reach the intervention/selected households.
6. Place in a requisition for water storage vessels with a spigot and lid. No filter is needed.
7. The required number of water storage vessels should be equal to the number of households surveyed in the intervention community.
8. The drinking water storage vessels should be appropriately cleaned. (See cleaning protocol)
9. After cleaning and drying, place lids on all the vessels.
10. Carefully place each vessel in a transparent rubber to prevent possible dust and introduction of foreign material during transportation.
11. Make the necessary transportation arrangements prior to the day of distribution.
12. The time period between cleaning and distribution should be no more than 1 week.
13. **Distribution phase.**
14. Inform the village leader or appropriate representative about the intended visit and the selective distribution.
15. Print out the GPS location of the selected households and the corresponding names of respondents.
16. Transport the storage vessels to the selected community.
17. Pay a courtesy call on the chief explaining the rationale behind the selective distribution.
18. Use a GPS device to locate selected households.
19. Confirm household location by looking out for the household code.
20. At the household, greet appropriately and provide the vessel to the respondent with detailed instructions (See: Instructions to households).
21. In each selected household, physically use the vessel to demonstrate how to fill, how to dispense water and how to minimize strain on the tap when dispensing water. Allow the recipient to practice fetching water in your presence.
22. When done with the distribution, return to say thank you to the village leader or representative and the Chief of the community.

# Adaptations

## First PDSA cycle

Two modifications were made to the prototype safe water storage container tested during the first PDSA cycle prior to implementation in the second cycle.

The first modification was to the hook on the lid of the water storage container, which was too large and did not allow for a firm closure of lid. However, the manufacturer of the container could not find a smaller hook on the market to improve the closure of the container. As a result, the manufacturer modified the existing hooks by bending their shape (Figure 4).

The second modification was to the interior guard on the container. The original guard was made of two crossed metal bars to prevent drawing of water directly from the container using another smaller container (e.g., cup or gourd) and required users of the container to draw water using the tap provided for this purpose. However, the edges of this metal guard injured the arms while cleaning the containers. The guard was modified to a single plastic crossbar to allow it to be cleaned and filled without injury.


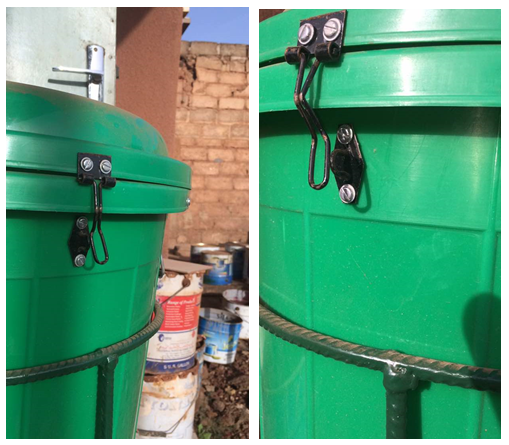


Figure 4. Original latch design shown at left, and modified design at right, with latch bent to improve a tighter, better fitting closure.


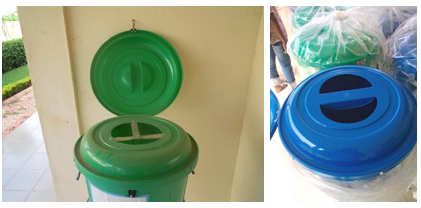


Figure 5. Original lid design shown at left and modified design at right, with interior guard made of plastic to reduce sharp edges.
