# Supplementary material for: Adapting a safe water storage container to improve household stored water quality in rural Burkina Faso: a cluster randomized trial
Source: J Water Sanit Hyg Dev. Author manuscript; Available in PMC 2025 Sep 23. (PMC12453111; doi:10.2166/washdev.2021.065)
Supplement: SI file 1 [file NIHMS2111177-supplement-SI_file_1.docx]

**WaterAid – CQI Project: Focus Group Guide**

**Purpose**: The purpose of these focus groups is to learn about current household water storage practices and to learn what households think of our potential designs for safe water storage containers. Before moving forward with a design, we want to learn what they like about them and what they do not like about them so we can make changes. Our goal is to create a safe water storage container that is effective in keeping water safe, and accepted and used by households.

**Timeline:**

- Each focus group should have 8–10 people so it may take about 2–3 hours to identify and recruit participants.
- On that day of the focus group, the Moderator and Assistant should show up ahead of time to make sure chairs are set up, the voice recorder works, and the Assistant has a notebook and pen.
- The focus group itself should last 1–1.5 hours. As a moderator, you will need to complete the focus group with all of the questions in 1.5 hours.
- After the group is over the Moderator and Assistant should meet for 30–60 minutes to review the notes taken and write down any additional observations or notes that were important.
- The WaterAid staff will take the containers, voice recorder and notes with them back to the WaterAid office.

**Recruiting Participants:**

WaterAid staff have identified the villages where we are going to conduct the focus groups. Each focus group should have 8–10 people. Some people may not show up, so it is good to invite 1–2 extra. Here are some characteristics to consider:

- **Potential customers**: Each person should be someone that currently stores water in their home
- **Representative**: In each village, we want to have a representative group, so look for people of different income levels, people from different groups
- **Genders**: We would like to have 6–7 women and 3–4 men
- **Ages**: Only recruit adults, but ask a range of ages amongst adults

**When asking people to participate, read the Focus Group Consent Form first (included at the end of this guide), and if they agree to participate then they can join the group**.

**Tools:**

When preparing for the focus group make sure you have:

- Pen and notebook
- Voice recorder and spare batteries
- Storage containers (Tall design and Jerry Can design)
- A copy of this guide

**Preparing for the focus group:**

1. Arrive early and set up chairs, voice recorder
2. Smile and welcome participants
3. Introduce yourself and let them know when the group will begin

**When it is time to start:**

Hello, my name is [ NAME ] and I work with [ partner organization name ] and we are partnering with WaterAid Burkina Faso and the University of North Carolina to improve household water quality. Thank you all for agreeing to participate in this focus group. We conducted a study with some households in your village to learn about household water storage practices. We found that many storage containers do not do a good job of keeping water clean. Therefore, we designed some new containers that will keep water safer. Today, I want to talk with you about your experience storing water in your homes and share our new container designs with you to see what you think. We will be audio recording this discussion to make sure we do not miss any responses.

Before we start, I want to share some ground rules:

1. [ NAME ] will be taking notes throughout the discussion
2. There are no wrong answers, so please express your opinion honestly
3. Please talk one at a time and in a clear voice and please avoid side conversations
4. You may disagree with others in the group but please be respectful and listen to one another
5. Feel free to address each other during the discussion, however only use first names
6. Stay the whole time – added in

I am now going to start the voice recorder so I can remember all of what you share.

1. **Intro**: **We would like to know what your current experience has been storing drinking water at your house.**
   1. Describe how you use drinking water storage containers currently

*Probe*: Where are they located?

*Probe*: How big are they (liters)?

*Probe*: What are their main characteristics (lids, taps, colors, material)

*Probe*: How do you get water from the container?

- 1. What do you like about your current drinking water container?
  2. What do you not like about it?
  3. Think back to when you purchased your drinking water storage container, why did you buy it? How much did you pay for your container?

1. **Thank you for sharing about your current experience storing water at your house. Now I would like to show you our first design and hear your thoughts about it.** *The moderator should now introduce the Tall Design and demonstrate to the group how to use the container. Then allow the group to try using it.*
2. How does this container compare to your current drinking water container?
3. What do you like about the Tall Design?
4. What would you change about Tall Design?
5. How do you think you would use the Tall Design?
6. Would you be likely to use this storage container instead of your current drinking water storage container? Why or why not?
7. **Thank you for your thoughts about the first design. Now I would like to introduce our second design.** *The moderator should now introduce the Jerry Can Design and demonstrate to the group how to use the container. Then allow the group to try using it.*
8. How does this container compare to your current drinking water container?
9. What do you like about the Jerry Can Design?
10. What would you change about Jerry Can Design?
11. How would you use the Jerry Can design?
12. Would you be likely to use this storage container instead of your current drinking water storage container? Why or why not?
13. **Now I want to put them side by side and ask a few more questions. When answering please refer to each design by “Tall Design” or “Jerry Can Design”.** *The moderator should make sure each design is visible to the entire group, and clearly labeled “Tall Design” and “Jerry Can Design”.*
    1. Would you buy either of the designs? If so, which one would you buy?
    2. Why would you choose to purchase that design? Or why would you not?
    3. How much would you be willing to pay for that container?
    4. Could you see yourself using the container throughout the year?

*If time remains in the focus group, then you can continue with these questions, but make sure that you have covered all the questions above.*

- 1. What trade name would you propose for each design?
  2. Now if you could design your ideal water storage container, what would it look like and what characteristics would it have?

Is there anything else you would like to share or questions you would like to ask?

**Summarize the major themes that you heard in the discussion and answer any additional questions before closing.**

**CLOSING**

On behalf of WaterAid Burkina Faso, the Water Institute at UNC and our partners, we thank you for participating in this focus group discussion. Your feedback is valuable in helping us improve the designs of our household water storage containers and in improving the safety of household stored water.

Again, please be assured that your name will not be associated with the responses you provided during the discussions. Only major themes and illustrative quotes that came from our discussion will be shared with other people.

Once again thanks for your time and participation.

**After the focus group:**

- The Moderator and Assistant should review notes and add any comments or observations that are missing
- The WaterAid staff member will take the voice recorder, notes, and containers with them back to the WaterAid office. Be sure to give the notes and voice recorder to Hermann Kambou.

**Tips for Moderators:**

**Neutrality**

- Neutrality is especially important in focus groups because we don’t want to influence their opinions.
- You want to remain as neutral or impartial as possible, even if you have a strong opinion about something that is said. Use phrases such as “Thank you. That is helpful,” or “I see.”
- Comments such as “I can’t believe it!” or “You really think that?!” reveal your opinion, which can negatively influence discussion.

**Challenges**

In focus groups, it can be common for a few individuals to dominate the discussion. In mixed gender groups, one gender may tend to speak more than the other. We want to hear from everyone in the group. To balance participation, and ensure that every participant has an opportunity to contribute, consider the following strategies:

- **The dominator and the “know-it-all”:** Acknowledge their contribution by saying, “Thank you. I appreciate your comments.” Then make direct eye contact with other people and say, “I’m interested in hearing what other people think about this question.”
- **The rambler:** Stop eye contact, stop taking notes, look at your watch, and jump in when they pause to take a breath.
- **The shy participant:** Make eye contact, smile at them, and address questions to them like, “(Name), I am curious what you think about that?”
- **The participant who talks very quietly:** Ask them to repeat their response more loudly.

**Off-topic conversations**

Sometimes the conversation will start to stray away from the topic of the focus group.

- When this happens, take advantage of a pause and say, “Thank you for that interesting idea. I think that because we are only covering certain topics today, we should discuss that at a different time.”
- You can always acknowledge that you only have a limited amount of time, and in order to hear from everyone and cover the rest of the questions, it is time to move on.

**Silence**

- If no one responds and the question may have been difficult to understand, try asking the question in a different way.
- If no one responds to a question, and you aren’t sure what the problem is, it’s okay sometimes to wait it out. Allowing silence at times encourages elaboration by participants because it gives them a chance to think about what they want to say.
- More often than not, participants will fill the silence with more information. It is important to balance keeping the conversation moving and allowing participants time to think and share.

**Listening**

Some behaviors show that you are engaged and keep participants engaged. Show participants that you are listening by:

- Leaning forward slightly
- Looking directly at participants while they are speaking
- Nodding at appropriate times
- Saying “mm-hmm” in response to what they say

Some behaviors show that you may not be listening and can cause participants to take their role of sharing expert knowledge less seriously. This could cause participants not to elaborate or provide detail. Avoid these behaviors:

- Looking away
- Yawning
- Frequently checking your watch
- Interrupting participants in a rude manner, or without explaining why you had to interrupt

**Keep them talking**

Generally, you want to interrupt participants as little as possible. If you feel that you need to follow-up with something they said, make a note of it and ask them about it when they have finished their thought.

**Probes**

Probes and clarifying questions are important to clarify what a participant has said and get more thorough responses. You should use probes for more detail about what the participant thinks, feels, and experiences when:

- A participant’s response or contribution is brief or unclear
- When a participant or the group seems to be waiting for a reaction from you before continuing to speak
- When a person appears to have more information on the subject

Examples

Some examples of general probes include:

- Please tell me more about that …
- Could you explain what you mean by that?
- Can you tell me something else about that?

**Note Taking**

**Note taking is a primary responsibility of the assistant moderator**

The moderator should not be expected to take written notes during the discussion.

**Clarity and consistency of note taking**

Anticipate that others will read and use your notes. Notes sometimes are interpreted days or weeks following the focus group when memory has faded.

**Tips for note taking**

1. Write down in your notebook:
   1. Date of the focus group
   2. Your name
   3. The moderator name
   4. Number of focus group members
   5. Village name where the focus group is taking place
2. Make a seating chart and letter the participants to keep track of who said what
3. When taking notes write the question number clearly and write the responses in order of how they were said with the participant letter next to who said it.
4. Separate your notes into participants responses and observations
5. Write down the participants quotes, write down the key phrases and words that they used.
6. Clarify responses before you write them down if you do not understand what they mean.
7. If anything unusual happens that affects the focus group, write it as an observation.
8. Write down any insights you have as a moderator with your initials beside your comment.
9. Take notes of any body language (head nods, physical excitement, eye contact) of participants that would indicate agreement with a thought shared by someone else.
10. After the focus group meet with the moderator and review the notes. Add any additional observations or comments that were not already written.

**Focus group consent form**

**Introduction and study purpose**:

Hello, I am [insert name] and I work with [insert partner organization name] who is partnering with WaterAid Burkina Faso and the Water Institute at the University of North Carolina to improve the safety of water that is collected and stored in containers by households. As part of the project, we have conducted a study to identify water storage container characteristics that improve the safety of water. Based on our results, we have identified and designed a few potential storage containers to be used by households. In order to learn about users’ opinions of the different designs, we are conducting eight focus groups of 8–10 people each. We have selected because you have experience storing water in your home and we would like to ask this group a few questions as part of our research. We will use your responses to determine which design is preferred by households and to identify any changes that need to be made to the design. The focus group will take approximately 1 to 1.5 hours.

Confidentiality and withdrawal

There are no right or wrong answers to the questions. The responses you provide during the focus group discussions will be kept confidential by the staff. Your name and any identifiable information will not be recorded, but we will assign you a number that we will use in the transcripts. We will record your responses and the discussion will be audio-recorded to make sure that we do not miss anything you have said. You can choose not to answer any of the questions or leave the discussion at any time.

Benefits and risks

There are no anticipated risks in participating in this discussion. You will not receive any compensation for your participation, however your responses will be used to help us design a product that will improve the quality of household stored water. Therefore, we would be grateful if you could answer as many questions as possible to the best of your ability and with your honest opinion.

Would you like to participate in our focus group?

[*If they agree give them details as to when the group is meeting; if they do not wish to participate, thank them and move on*]

Thank you for your time and willingness to contribute to these discussions. The ethics committee of the University of North Carolina has approved this project and the Ethics Committee of Ministry of Health has given permission to carry out these interviews. Please feel free to contact Hermann Kambou at the WaterAid Office in Ouagadougou if you have any issues or concerns in participating in the focus group.

Hermann Kambou

WaterAid office +226 70 12 01 60
